# Genome-wide association study and genomic prediction using parental and breeding populations of Japanese pear (*Pyrus pyrifolia* Nakai)

**DOI:** 10.1038/s41598-018-30154-w

**Published:** 2018-08-10

**Authors:** Mai F. Minamikawa, Norio Takada, Shingo Terakami, Toshihiro Saito, Akio Onogi, Hiromi Kajiya-Kanegae, Takeshi Hayashi, Toshiya Yamamoto, Hiroyoshi Iwata

**Affiliations:** 1 Laboratory of Biometry and Bioinformatics, Department of Agricultural and Environmental Biology, Graduate School of Agricultural and Life Sciences, The University of Tokyo, 1-1-1 Yayoi, Bunkyo, Tokyo, 113-8657 Japan; 2 Institute of Fruit Tree and Tea Science, National Agriculture and Food Research Organization (NARO), 2-1 Fujimoto, Tsukuba, Ibaraki, 305-8605 Japan; 3 Institute of Crop Science, NARO, 2-1-2 Kannondai, Tsukuba, Ibaraki, 305-8518 Japan

## Abstract

Breeding of fruit trees is hindered by their large size and long juvenile period. Genome-wide association study (GWAS) and genomic selection (GS) are promising methods for circumventing this hindrance, but preparing new large datasets for these methods may not always be practical. Here, we evaluated the potential of breeding populations evaluated routinely in breeding programs for GWAS and GS. We used a pear parental population of 86 varieties and breeding populations of 765 trees from 16 full-sib families, which were phenotyped for 18 traits and genotyped for 1,506 single nucleotide polymorphisms (SNPs). The power of GWAS and accuracy of genomic prediction were improved when we combined data from the breeding populations and the parental population. The accuracy of genomic prediction was improved further when full-sib data of the target family were available. The results suggest that phenotype data collected in breeding programs can be beneficial for GWAS and GS when they are combined with genome-wide marker data. The potential of GWAS and GS will be further extended if we can build a system for routine collection of the phenotype and marker genotype data for breeding populations.

## Introduction

Breeding of fruit trees is a long-term process because of their long juvenile period^1^. Pears, which are important fruit tree species^2^ in the genus *Pyrus* (Rosaceae, Pyrinae), have a juvenile period of 6–12 years^1^. ‘Kosui’ and ‘Hosui’, major cultivars of Japanese pear (*Pyrus pyrifolia* Nakai) were released 18 years after the parental cultivars were crossed^3^. Marker-assisted selection (MAS), especially at an early seedling stage, is a promising strategy for improving the efficiency of selection in fruit tree breeding, because it enables the selection of a large number of individuals, accelerates selection and crossing, and reduces cultivation cost. However, MAS has not been widely used in fruit tree breeding, especially in the improvement of fruit yield and quality traits, because of some technical limitations^4,5^. Bi-parental quantitative trait locus (QTL) mapping for detecting QTL-linked markers is time- and cost-consuming, especially in the preparation of an experimental population, and an expected marker effect may not be attained in a different genetic background^6^. An intrinsic problem of MAS is that it is not suitable for the improvement of complex traits controlled by a number of genes^4,7^.

High-throughput genotyping technologies have greatly reduced the cost and time of genotyping and have made a large number of markers routinely available, which enables us to use genome-wide association study (GWAS) and genomic selection (GS) to overcome the limitations of MAS^5,8^. GWAS enables the detection of QTLs or causal genes for a target trait without using a bi-parental segregating population^9^. GS enables the selection of superior individuals based on genomic estimated breeding values, which take into account the effects of multiple genes controlling a target trait^4,10^. Combining GWAS and GS with MAS will accelerate breeding cycles^11^ and rationalize the design of breeding programs^12,13^.

Although the power and resolution of GWAS^14^ and the accuracy of GS^15^ are generally improved by large datasets, preparation of such datasets is sometimes not practical because of the characteristics of fruit trees mentioned above. Routinely collected breeding population data could be useful in GWAS and GS of fruit trees and beneficial for functional plant genomics^16,17^. A previous study that used GWAS and GS for parental varieties of Japanese pear revealed the potential of these approaches^12^. However, the statistical power of GWAS was rather low because the phenotypic values of the varieties were characterized as ordinal categorical scores^12^. The full potential of GWAS and GS needs to be assessed using not only parental populations, but also continuously evaluated practical breeding populations.

In the present study, we evaluated the power of GWAS and accuracy of genomic prediction using a parental population of 86 varieties and breeding populations of 765 trees from 16 full-sib families that have been evaluated in routine breeding. Our objective was to use GWAS to find the candidate genomic regions for the fruit quality traits and to validate the optimal model for GS in the breeding populations. Finally, we discuss the potential of using breeding populations evaluated routinely in breeding programs for GWAS and GS in fruit tree breeding.

## Results

### Linkage disequilibrium decay and population structure

**Figure 1:**
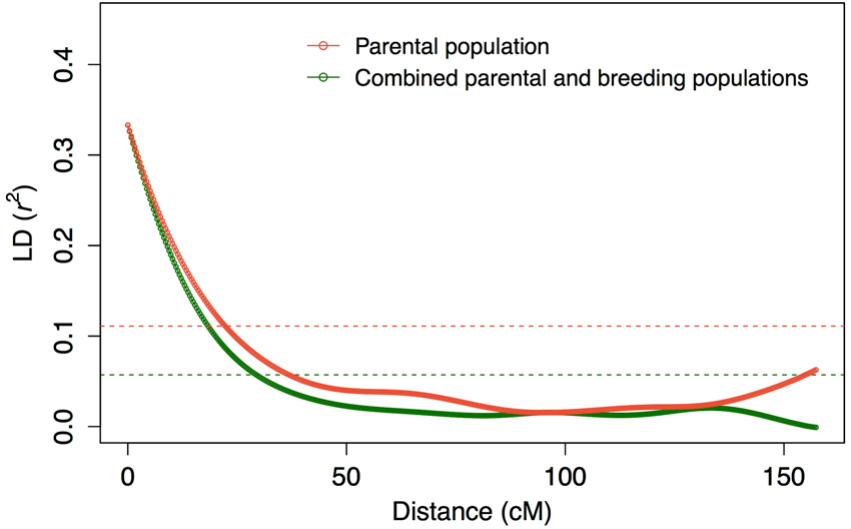
LD decay estimated from parental and combined populations. Curves show local polynomial smoothed plots with kernel weight for the parental population (n = 86) and combined parental and breeding populations (n = 851). Horizontal dashed lines represent the baseline *r*^2^ values based on the 95^th^ percentile of the distribution of *r*^2^ values between pairs of unlinked markers.

The curves fitted for the relationships between linkage disequilibrium (LD) *r*^2^ values and linkage map distances (Supplementary Fig. S1; Supplementary Methods) showed that high degrees of LD extended over 20 cM in both populations (Fig. 1; Supplementary Tables S1 and S2). For markers 10 cM and 20 cM apart, the *r*^2^ values were 0.20 and 0.12, respectively, in the parental population, and 0.18 and 0.10 in the combined population. The decay of LD was slightly faster in the combined population than in the parental population. The mean *r*^2^ value between adjacent SNPs was higher in the combined population (0.34) than in the parental population (0.33). In contrast, the 95^th^ percentile of *r*^2^ values between unlinked SNPs (i.e., SNPs located on different linkage groups (LGs)) was higher in the parental population (0.11) than in the combined population (0.05). The patterns of LD decay were almost identical in all LGs except for LG 16 (Supplementary Fig. S2). The LD on LG 16 decayed slowly in both populations.

**Figure 2:**
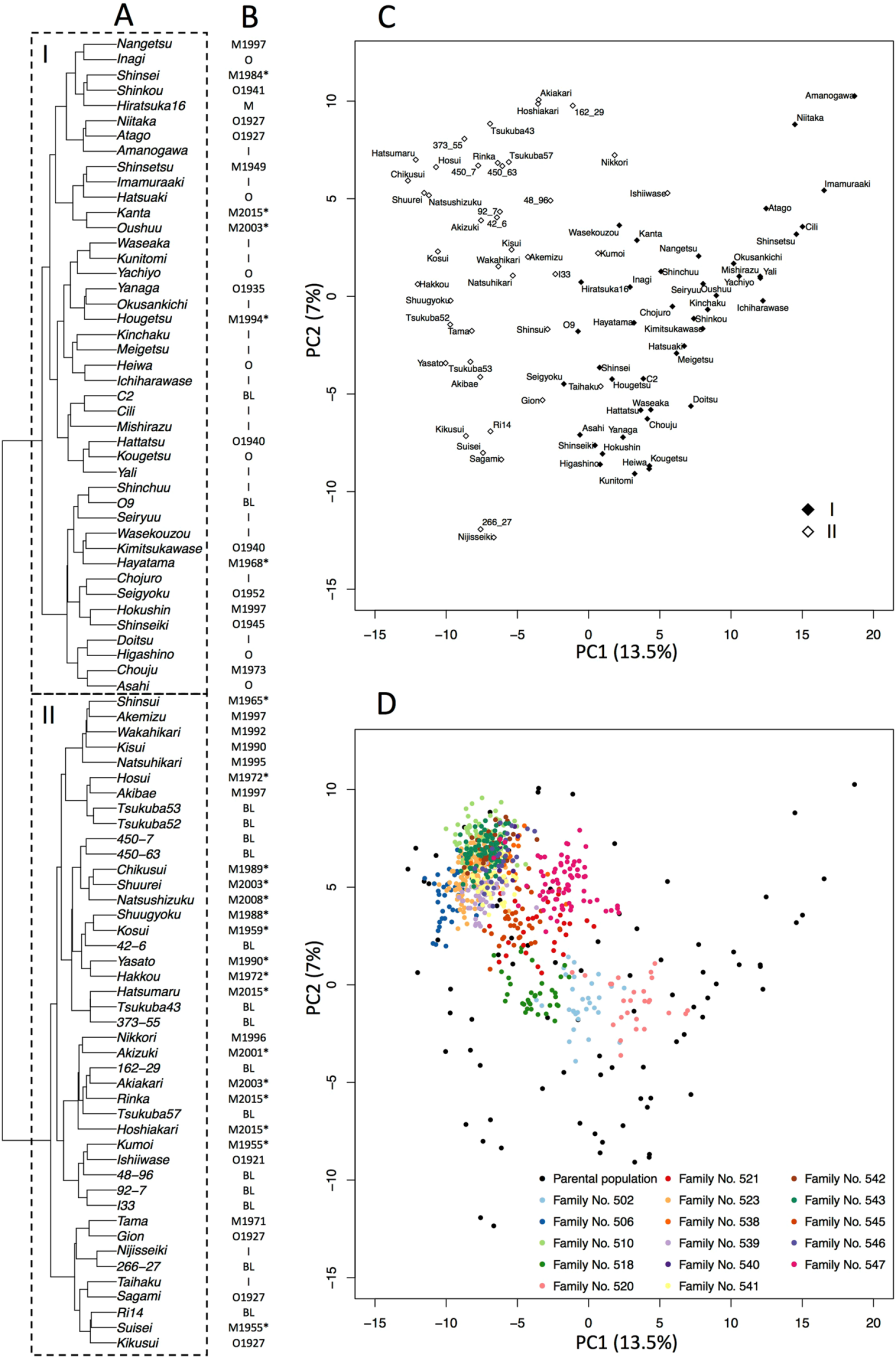
Structures of parental and combined populations. (**A**) Hierarchical clustering of the parental population. (**B**) Types of varieties (I, indigenous; O, old; M, modern; BL, breeding line) and release years (Supplementary Table S1). Asterisks indicate modern elite cultivars bred by the NARO Institute of Fruit Tree Science. (**C**) PCA of the parental population. Black and white diamonds indicate clusters I and II estimated by hierarchical clustering, respectively. (**D**) PCA of the combined population. Black circles indicate the parental population. Coloured circles indicate members of each family of the breeding population (see Supplementary Table S2).

Hierarchical clustering indicated that the 86 varieties in the parental population fell into two major clusters (Fig. 2A), which mainly contained indigenous/old (Cluster I) or modern cultivars (Cluster II) (Fig. 2B). Although two clusters were also revealed by principal component analysis (PCA) plots of the parental population (Fig. 2C), the structure of these clusters was ambiguous. Small ambiguous clusters, each corresponding to an F_1_ family, were observed in the combined population (Fig. 2D). Some of the clusters were located around modern elite cultivars, such as ‘Kosui’, ‘Hosui’ and ‘Akizuki’ (Fig. 2C and D).

### GWAS

**Figure 3:**
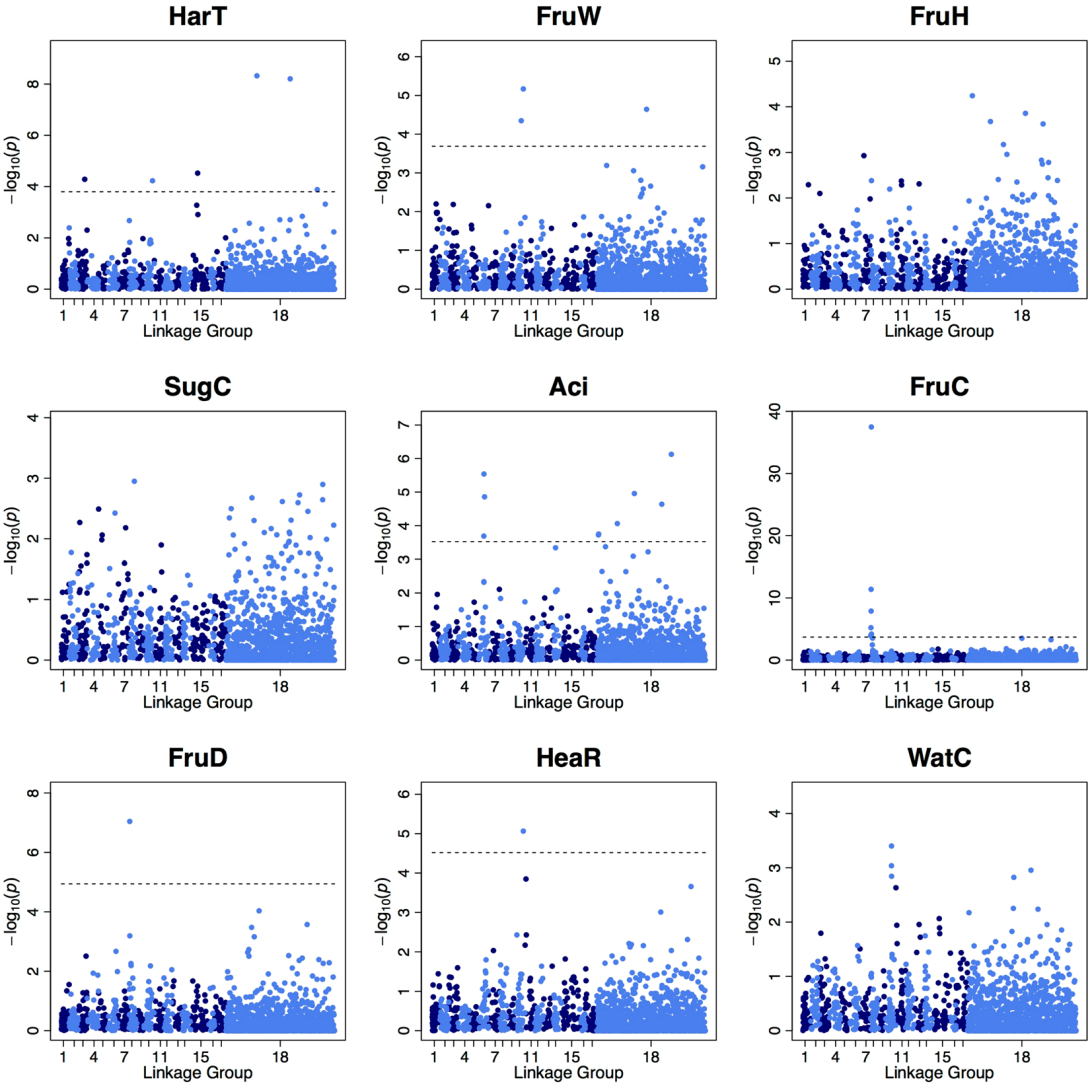
Manhattan plots for nine fruit quality traits in the single-locus GWAS using combined population. Dashed lines indicate a false discovery rate of 0.05. Linkage group 18 is a fictive linkage group for placing SNPs not mapped on 17 linkage groups.

One single-locus and three multi-locus GWASs were conducted for 18 traits in the parental population (Supplementary Fig. S3; Supplementary Tables S3 and S5) and for 9 of these traits (harvest time (HarT), fruit weight (FruW), flesh firmness (FruH), sugar content (SugC), acid content (Aci), fruit skin colour (FruC), preharvest fruit drop (FruD), heart rot (HeaR), and watercore (WatC)) in the combined population (Fig. 3; Supplementary Tables S4 and S6). Higher –log_10_(*p*) values and/or more significant SNPs (false discovery rate (FDR) <0.05) were detected for all the fruit quality traits in the single-locus GWAS using the combined population than in the parental population. More significant SNPs (critical logarithm of odds (LOD) score ≥3) were also detected in the combined population than in the parental population in the multi-locus GWAS methods. The number of significant SNPs detected in the multi-locus GWAS methods was larger than in the single-locus GWAS, and ISIS EM-BLASSO detected the largest number of the significant SNPs among the three multi-locus GWAS approaches. Significant SNPs in the single-locus GWAS were detected for five traits (Aci, FruC, FruD, resistance to black spot 1 (BSR1), and BSR2) in the parental population (Supplementary Table S3) and for six traits (HarT, FruW, Aci, FruC, FruD, and HeaR) in the combined population (Supplementary Table S4). Two common significant SNPs were detected for BSR1 and BSR2 in the single-locus GWAS using the parental population (LG 18; phenotypic correlation (*r*) = 0.93; Supplementary Table S7), and the one of the two SNPs was also significantly detected for the both traits in two multi-locus GWASs (FASTmrEMMA and ISIS EM-BLASSO; Supplementary Table S5). A significant SNP detected for FruC corresponded to the largest (but non-significant) peak SNP for rust (Rust) in the single-locus GWAS using the parental population (LG 8; *r* = −0.67). The corresponding SNP for FruC and Rust was significantly detected for both traits in the multi-locus GWAS (FASTmrEMMA). Single common significant SNPs were detected for HarT, FruW, and HeaR (LG 10; *r* = 0.65 for HarT and FruW) and for FruC and FruD (LG 8; *r* = −0.42) in the single-locus GWAS using the combined population. Common significant SNPs for HarT, FruW, and HeaR, and FruC and FruD were also significantly detected in two (FASTmrEMMA and ISIS EM-BLASSO) or one (FASTmrEMMA) multi-locus GWASs, respectively (Supplementary Table S6).

### Single-trait genomic prediction

**Figure 4:**
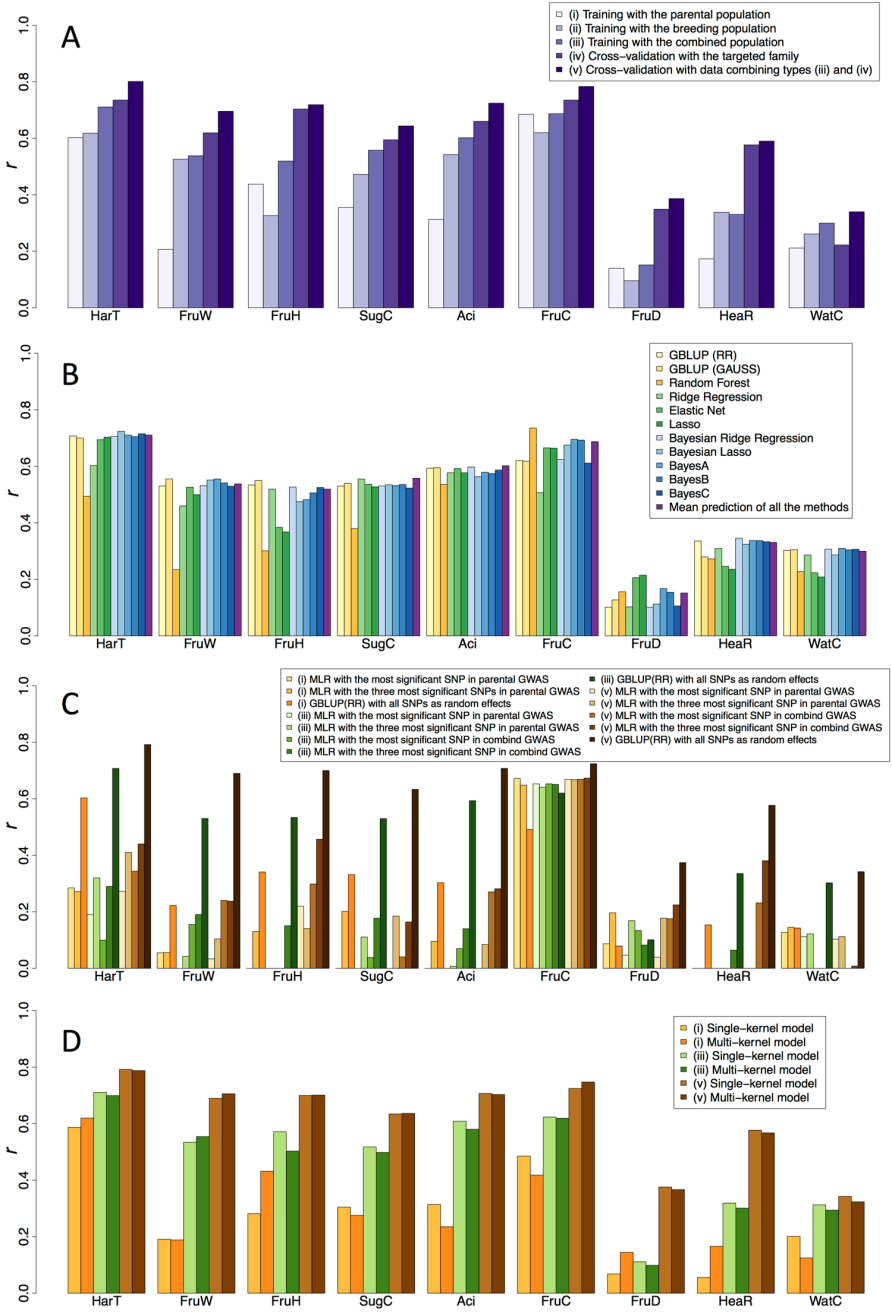
Prediction accuracy of single-trait models for the breeding populations. Prediction accuracy was measured as Pearson’s correlation coefficient (*r*) between predicted genotypic values and phenotypic values. The prediction accuracy was calculated for all families combined. (**A**) Five types of validation were compared. Only the mean prediction accuracy of all methods (**B**) is shown. (**B**) Twelve methods were tested. RR: ridge kernel regression, GAUSS: Gaussian kernel regression. Validation of type (iii) is shown (**A**); other validation types are shown in Supplementary Fig. S6. (**C**) Regression models were based on the results of the single-locus GWAS using parental or combined population. One or three SNPs that showed high −log_10_(*p*) values in GWAS were selected for MLR. (i), (iii), and (v) indicate validation types (**A**). MLR: multiple linear regression. (**D**) Prediction models that considered only additive or both additive and dominance effects were tested.

To evaluate the accuracy of genomic prediction for families in the breeding population, five types of validation were used. For eight traits except HeaR, higher prediction accuracy was attained in the combined population as a training set (type iii) than in the parental population (type i) or breeding populations as training sets (type ii) (Fig. 4A). When prediction models were trained with data from a family targeted by genomic prediction (10-fold cross-validation (CV) with the targeted family (type iv), and 10-fold CV with combined type (iii) and (iv) data (type v)), the prediction accuracy was further improved (Fig. 4A). The greatest accuracy was attained in type (v) validation. Similar trends were observed for single families (Supplementary Figs S4 and S5). In type (v) validation, the prediction accuracy was high for HarT, FruW, FruH, Aci, and FruC (*r* ≥ 0.7), intermediate for SugC and HeaR (0.5 ≤ *r* < 0.7), and low for FruD and WatC (*r* < 0.5) (Fig. 4A). For FruC, the upper-middle level of prediction accuracy (*r* = 0.68) was attained even in type (i) validation.

Accuracy of genomic prediction was compared among 12 methods for the breeding populations. The accuracy of Random Forest exhibited large differences depending on the trait, whereas other methods exhibited smaller differences or were relatively stable among the methods (Figs 4B and S6). The mean of 11 methods always showed an upper-middle level of accuracy among the methods, for all traits. Similar results were obtained for single families (Supplementary Figs S7 and S8). For FruC, Random Forest attained the greatest accuracy in the breeding populations (Figs 4B and S6). Random Forest, however, was not the most accurate method in seven families (510, 518, 538, 541, 543, 546, and 547) in single-family-based type (iii) evaluation (Supplementary Figs S7 and S8). In all of these seven families except 543, the within-family phenotypic variation was biased toward either smooth (green) or russet (red) skin (Supplementary Figs S9 and S10; Table 1). In families 518 and 543, no polymorphism was observed in the most significant SNP allele (Supplementary Fig. S9). For Rust, Random Forest was most accurate in CV using the parental population (Supplementary Fig. S11A).

**Table 1:** Phenotypic traits evaluated in this study.

Trait	Abbreviation	Continuous or categorical value	Number of levels	Description	Rate of missing value in parental population	Rate of missing value in combined population
Harvest time	HarT	Continuous	—	Number of days to harvest from July 1st	0	0
Fruit weight	FruW	Continuous	—	Mature Fruit weight (g)	0	0
Flesh firmness	FruH	Continuous	—	Magness-Taylor pressure test (lb)	0.01	0.004
Sugar content	SugC	Continuous	—	Total soluble solid content of juice (%)	0.01	0.004
Acid content	Aci	Continuous	—	pH of juice	0.01	0.004
Fruit skin color	FruC	Categorical	5	Smooth (russet formation on 0–20% of the surface area of mature fruit), smooth (20–75%), smooth (75–95%) middle (95–99%), russet (100%) (visual)	0.02	0.005
Preharvest fruit drop	FruD	Continuous	—	Ratio of preharvest fruit drop (visual)	0	0
Heart rot	HeaR	Continuous	—	Ratio of heart rot (visual)	0.01	0.004
Watercore	WatC	Continuous	—	Ratio of watercore (visual)	0.01	0.004
Severe watercore	SWatC	Continuous	—	Ratio of severe watercore (visual)	0.03	—
Fruit shape in longitudinal section	FruS	Categorical	5	Round, oblate, broad elliptical, oval, obovate (visual)	0.05	—
Rust	Rust	Categorical	4	None, a few, intermediate, many (visual)	0.07	—
Appearance	Appear	Categorical	5	Very bad, bad, intermediate, good, very good (sensory)	0.09	—
Groove	Groove	Categorical	3	None, a few, many (visual)	0.08	—
Resistance to black spot 1	BSR1	Categorical	3	Weak, intermediate, strong (visual)	0.02	—
Resistance to black spot 2	BSR2	Categorical	2	Susceptibility, resistance (visual)	0.02	—
Vigor of tree	TreV	Categorical	3	Weak, intermediate, strong (visual)	0.02	—
Number of spurs	SpuN	Categorical	3	few, intermediate, many (visual)	0.02	—

GBLUP (RR) with all SNPs outperformed multiple linear regression (MLR), with significant SNPs detected by single-locus GWAS in the combined population for all nine traits in type (v) validation (Fig. 4C). For FruC, the difference between GBLUP and MLR was small, and the upper-middle level of prediction accuracy (*r* > 0.65) was observed even in MLR. The accuracy tended to be higher for MLR based on GWAS in the combined population than for MLR based on GWAS in the parental population for traits in which the power of GWAS was higher in the combined population than in the parental population. For Rust, BSR1, and BSR2, the MLR model showed high accuracy in CV using the parental population (Supplementary Fig. S11B).

For all nine traits in type (v) validation, the prediction accuracy of the multi-kernel model, which considered both dominance and additive genetic effects, was almost the same as that of the single-kernel model, which considered only additive effects (Fig. 4D). Additive genetic effects were the major factor contributing to genetic variation for the six traits except FruD (Supplementary Table S8). For Appear, the multi-kernel model was more accurate than the single-kernel model in CV using the parental population (Supplementary Fig. S11C), and the dominance genetic effects were larger than the additive genetic effects (Supplementary Table S9).

### Multi-trait genomic prediction

**Figure 5:**
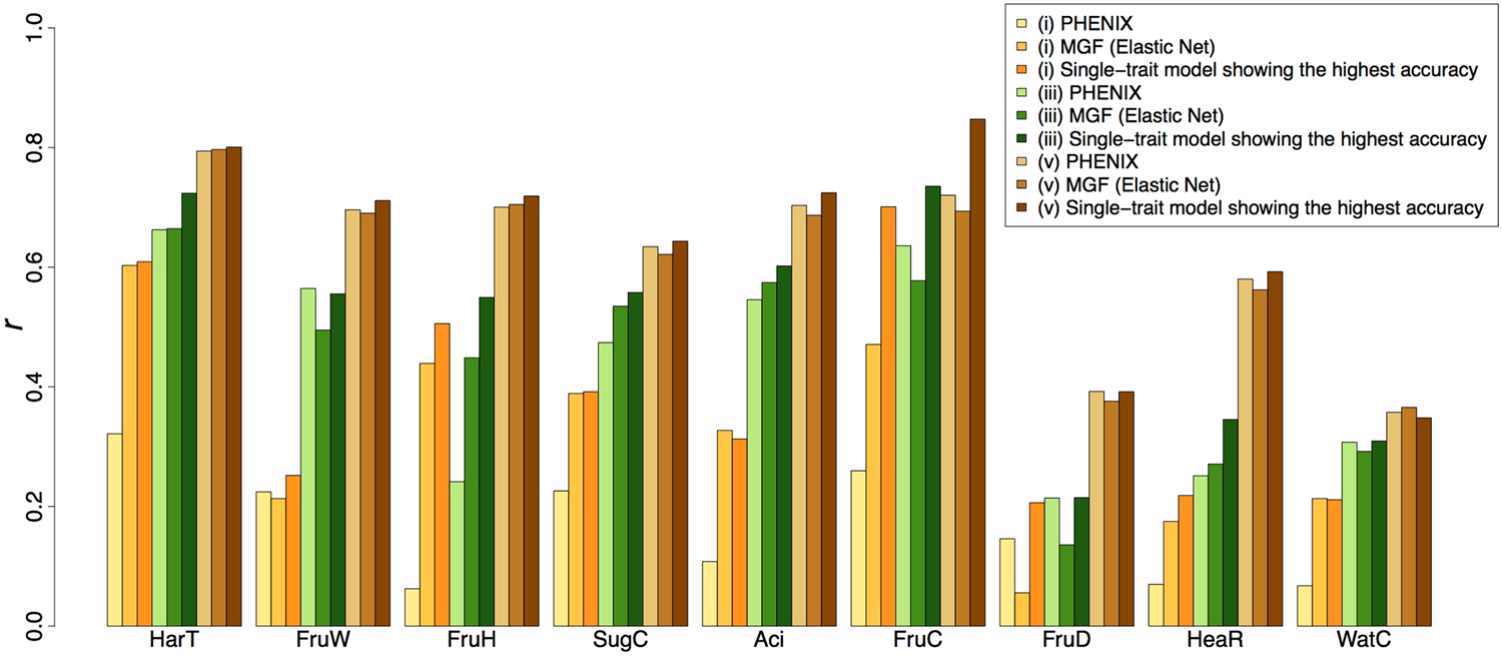
Comparison of single- and multi-trait models for the breeding populations. Prediction accuracy was measured as Pearson’s correlation coefficient (*r*) between predicted genotypic values and phenotypic values. (i), (iii), and (v) indicate validation types (Fig. 4A). PHENIX: Bayesian multivariate mixed model fitted via variational Bayes, MGF: multiple-response Gaussian family.

The difference in prediction accuracy between the multi- and single-trait models was small in type (v) validation for eight of the nine traits, the exception being FruC where the single-trait model outperformed the multi-trait model (Fig. 5). For fruit shape in longitudinal section (FruS) and number of spurs (SpuN), the multi-trait model was more accurate (*r* > 0.05) than the single-trait model in CV using the parental population (Supplementary Fig. S12).

## Discussion

LD reflects population genetic processes, such as mutation, recombination, the mating system, and the breeding system^18,19^. The resolution of GWAS and accuracy of GS depend on the pattern of LD^4,14^. In the present study, the wide range of LD in the parental population (*r*^2^ > 0.20 at 10 cM; Fig. 1) indicated a historical genetic bottleneck in this population, as suggested by Iwata *et al*.^12^. The mean *r*^2^ values between adjacent SNPs in the parental (0.33) and combined (0.34) populations were higher than the *r*^2^ value (0.2) that is necessary for accurate genomic prediction^20^, and were slightly higher than in other rosaceous species, apple (0.32)^21^ and strawberry (0.26)^22^.

Subpopulation structure in a target population causes spurious association in GWAS^14^ and influences the accuracy of genomic prediction^23^. The structure in the parental population was ambiguous, which may reflect the narrow genetic background of Japanese pear cultivars^24^. The LD between unlinked markers was lower in the combined population than in the parental population, suggesting that adding multiple segregating families to the parental population made subpopulation structure more ambiguous. Weak subpopulation structure in the parental and combined populations may improve the resolution of GWAS and accuracy of genomic prediction.

In single- and multi-locus GWASs, more significant SNPs (FDR <0.05 or LOD score ≥3) were detected for all nine fruit quality traits in the combined population than in the parental population, thus indicating that combining data from multiple families with the data of the parental population increases GWAS power. Combining multiple populations increased GWAS power in citrus^25^ and dairy cattle^26^. Meta-analysis of GWAS revealed the efficiency of combining multiple populations for pig^27^. The higher degree and shorter range of LD in combined populations may reduce false positives in GWAS^14,28^. Many QTLs are shared between closely related populations^26^ and may also increase the power of GWAS.

SNPs detected in GWASs may be useful as markers for MAS. For HarT, significant associations in single- and multi-locus (FASTmrEMMA) GWASs were detected on LGs 3, 10, and 15, and LGs 3 and 15 of them were consistent with associations detected previously^12^. On LGs 3 and 15, QTLs for harvest time were detected using a bi-parental segregating population derived from ‘Akiakari’ × ‘Taihaku’^29^. Chen *et al*.^30^ revealed that most of the common markers for Japanese pear and Chinese pear were mapped on corresponding LGs in the same order and at similar distances. However, the associations detected in this study are inconsistent with the fruit maturity date QTL detected in a population derived from two Chinese pear varieties, ‘Bayuehong’ and ‘Dangshansuli’^31^. In apple, harvest time QTLs were identified on LGs 3, 9, 10, and 16 in a segregating population derived from ‘Telamon’ and ‘Braeburn’^6^ and on LGs 3, 10, 15, and 16 in a population derived from ‘Orin’ and ‘Akane’^32^. Because the level of collinearity between chromosomes of pear and apple is high^33^, the results in apple and in the present study strongly suggest the presence of a harvest time QTL on LG 10 of Japanese pear.

On LG 15, one 1-aminocyclopropane-1-carboxylate (ACC) synthase gene (*PPACS2*) in Japanese pear^29^ and two ACC synthase genes (*MdACS1* and *MdACS3*) in apple^32^ have been mapped previously. ACC synthase catalyses the synthesis of the ethylene precursor ACC from S-adenosyl methionine. In Japanese pear^34^ and apple^35^, ripening and fruit storage potential are closely related to the amount of ethylene produced. The QTL for HarT on LG 15 is tightly linked to the preharvest fruit drop in apple^32^, and a significant association for FruD was observed on LG 15 in the multi-locus GWAS (mrMLM) of this study. The significant association on LG 10 for HarT was consistent with that for FruW and HeaR, suggesting pleiotropy or close linkage among QTLs for these traits, which resulted in the high phenotypic correlation between HarT and FruW. A high genetic correlation (*r* > 0.7) between ripening time and fruit weight was described by Abe *et al*.^36^. In a population derived from the Chinese pear varieties ‘Bayuehong’ and ‘Dangshansuli’, one of the four QTLs for fruit weight was located on LG 10^31^, consistent with the presence of a QTL for FruW on LG 10 in Japanese pear.

One large significant association for FruC on LG 8 both in single- and multi-locus (FASTmrEMMA) GWASs was consistent with an association mapping study of skin russet coverage in *Pyrus* spp.^37^. Yamamoto *et al*.^29^ identified one major QTL for fruit skin colour on LG 8. In GWAS using an apple population generated from a factorial mating design of four female and two male parents, a large association for skin russet coverage was observed on LG 1^21^. In a cross of ‘Renetta Grigia di Torriana’ × ‘Golden Delicious’ apple, a QTL for russet skin was mapped on LG 12^38^. Although QTLs for russet skin have been mapped on different LGs in pear and apple, ATP-binding cassette (ABC) transporters have been implicated in this trait in both pear^39^ and apple^38^. The major components of russet skin are lignin, cellulose, and hemicellulose^40^, and ABC transporters are involved in lignin, cutin, and suberin transport^41,42^. In Japanese pear, some ABC transporters displayed differences among russet- and green-pericarp genotypes in RNA-seq analysis^39^. The significant association on LG 8 for FruC was consistent with that for FruD in the single- and a multi-locus (FASTmrEMMA) GWASs using the combined population and with that for Rust in the multi-locus GWAS (FASTmrEMMA) using the parental population. The common association on LG 8 for these traits could imply pleiotropy of close linkage among QTLs controlling these traits, resulting in the high or moderate phenotypic correlations among the traits.

The largest (although not significant in the single-locus GWAS) peak SNP detected on LG 8 was for SugC, which was significant in a multi-locus GWAS (FASTmrEMMA), and was consistent with one of the QTLs detected in Japanese pear^29^. For Aci, a QTL on LG 14 detected in Japanese pear^29^ was consistent with the significant SNP detected in a multi-locus GWAS (ISIS EM-BLASSO), but not with the significant association on LG 6 detected in both single- and multi-locus (FASTmrEMMA and ISIS EM-BLASSO) GWASs.

A larger number of significant SNPs were detected in the multi-locus GWASs than in the single-locus GWAS. This implies that the multi-locus GWAS methods have larger power than the single-locus GWAS for the fruit quality traits evaluated in this study, because the traits are quantitative and are controlled by multiple, sometimes numerous, genes or QTLs. The advantage of multi-locus GWAS methods for complex traits controlled by multiple loci has been reported^43,44^. Many common significant SNPs were detected in both approaches, whereas some of the significant SNPs were not consistent between the one single-locus and three multi-locus GWAS methods, as mentioned above. This suggests that a combination of single- and multi-locus GWAS methods could increase the chances of identifying of genes or QTLs that control the traits.

In genomic prediction for the breeding population, training with the combined population attained higher prediction accuracy than training with the parental population or the breeding populations only. The result may imply that the parental and breeding populations are closely related to each other and that marker effects are almost the same across populations. In general, genomic prediction across populations that are of low relevance has lower accuracy than genomic prediction within a population, as discussed in an apple study^45^. However, higher accuracies have been obtained across a population based on combining multiple populations in a training dataset than within a population when multiple populations were closely related and marker effects were the same across both populations^23,46,47^. Because the collection of phenotypic data is not easy in fruit trees, genomic prediction using data from multiple breeding populations will be beneficial. The validity of using multiple breeding populations for genomic prediction has been described in citrus^25^. In the present study, genomic prediction was most accurate when models were trained with the data from the family targeted by GS because of a close relation between training and test sets and accurate estimation of marker effects.

Among the 12 methods for single-trait genomic prediction, Random Forest performed best for FruC in evaluation based on all families, whereas the accuracy of other methods showed small differences or were relatively stable among the methods. The mean of 11 methods always exhibited an upper-middle level of accuracy among the methods for all traits. This suggests that Random Forest is an appropriate method for FruC, whereas the mean of the methods exhibiting stable accuracy would be more suitable for the other traits, although the best method varies depending on the traits. The stability of the mean of all methods compared was also shown by Onogi *et al*.^48^. Random Forest is a machine learning method that can be effective in capturing large-effect QTLs^23,49^ and their interactions^48^. In rice, Random Forest was the best-performing model among GS methods for flowering time, in which a single large-effect QTL on Chr. 3 was identified by GWAS^49,50^. In our present study, GWAS detected a highly significant association on LG 8. As in a study by Spindel *et al*.^49^, the highly significant association may cause the superiority of Random Forest for FruC. On the other hand, Random Forest was not the best model for FruC in seven individual families, in which phenotypic variance was biased toward smooth green or red russet skin, and/or no polymorphism was observed in the most significant SNP. The result implies that the most significant SNP for FruC mainly explained the difference between green and red skin, but not variation within each colour. MLR with the most significant SNP also showed high prediction accuracy for FruC in the breeding populations, suggesting that traditional MAS with a marker for the significant SNP is useful in this trait. Because MLR with the most significant SNP was also accurate for Rust, BSR1, BSR2, and FruC in the parental population, traditional MAS may also be useful for these traits, especially for selection of parents.

For all nine traits, little difference was observed between the multi-kernel model considering dominance and additive genetic effects and the single-kernel model considering only additive effects. The result implies that the additive model was sufficient to explain genetic variation in the population, or the model that considers dominance and additive genetic effects could not be beneficial because of small dominance variation. In a study simulating *Eucalyptus* breeding^51^, inclusion of dominance effects improved the prediction of the total genotypic values in specific situations where the dominance-to-additive variance ratio (≥0.5) and broad-sense heritability (0.6) were high. As suggested by Zhao *et al*.^52^ and Denis and Bouvet^51^, a small population size might also reduce the benefit from a model considering dominance effects. Moreover, additive variance in the additive model tends to capture non-additive variation^53^, as experimentally confirmed^54^. This could also be a reason why the dominance model did not increase the accuracy of genomic prediction. In contrast, for Appear the multi-kernel model was more accurate than the single-kernel model. Dominance variance in Appear was predominant in the parental population. The result suggests that the dominance effect should be taken into account in the selection for Appear.

The accuracy of the multi-trait and single-trait models was almost the same for the eight traits other than FruC, thus suggesting that the multi-trait model would be beneficial for eight traits, because multi-trait models allow a prediction model to be simultaneously built for all traits. For FruC, however, the single-trait model should be used. Multi-trait models perform better than single-trait models when phenotypic data are not available for all individuals and traits^55,56^. A few missing values of phenotypes in a combined population can result in almost the same performance of these two models. In CV using the parental population, the prediction accuracy of multi-trait models was higher than that of the single-trait models for FruS and SpuN. The rate of missing values for FruS was comparatively high (Table 1), which increased the accuracy of the multi-trait model^55,56^. The continuous variation of FruS was difficult to evaluate because phenotyping was based on sensory or visual methods, which could also cause difficulties for prediction by the single-trait model. The multi-trait model may be very useful for practical breeding when data are missing because of natural disasters or human errors and trait evaluation is difficult.

In conclusion, the power and resolution of GWAS and the accuracy of GS were increased in a combined parental and breeding population. The prediction accuracy was further improved if the model included the information on a family targeted for GS. Our results suggest that phenotype data routinely collected for breeding populations can be useful for GWAS and GS when they are combined with genome-wide marker data. Accumulation and analysis of such data can increase the efficiency of breeding through MAS and GS and can contribute to detection and identification of genes responsible for complex traits by single- and multi-locus GWASs, which will further advance the functional genomics of fruit trees, as suggested by Poland^16^. Because large amounts of data cannot be accumulated in a short period of time for fruit trees, it is important to construct a streamlined system for routine collection and accumulation of the phenotype and genome-wide marker data from breeding populations.

## Methods

### Plant materials and phenotyping

As a parental population, we used 84 varieties of Japanese pear (*Pyrus pyrifolia* Nakai), which included 33 modern, 19 old, and 16 indigenous cultivars, and 16 breeding lines, of which 74 varieties were identical to those used in our previous study^12^, and 2 indigenous cultivars of Chinese pear (*P*. ×*bretschneideri* Rehd.) (Supplementary Table S1). As breeding populations, we used 16 full-sib families consisting of 765 F_1_ individuals in total (Supplementary Table S2). The breeding populations were derived from crosses among 18 Japanese pear varieties, all of which, except cultivar ‘Okuroku’, were included in the parental population. All plants were grown in experimental fields of the NARO Institute of Fruit Tree Science (Ibaraki, Japan).

In the parental population, 18 traits (14 fruit quality traits, 2 disease resistance traits, and 2 growth traits) were evaluated (Table 1). In the breeding populations, 9 out of 14 fruit quality traits were evaluated: harvest time (HarT), fruit weight (FruW), firmness of flesh (FruH), sugar content (SugC), acid content (Aci), fruit skin colour (FruC), preharvest fruit drop (FruD), heart rot (HeaR), and watercore (WatC). Fruits were sampled as described by Yamamoto *et al*.^29^. Several parental varieties were evaluated in 2013 and 2014, and the entire parental population was evaluated in 2015. Families 502, 506, 510, 518, 520, 521, and 523 (Supplementary Table S2) were evaluated in 2013–2015, and the remaining families were evaluated in 2014 and 2015. To remove the influence of the yearly effect, we fit a mixed linear model (MLM), in which the yearly effect was treated as fixed and the effect of genotype (tree) was treated as random. The best linear unbiased predictions (BLUP) of the genotype effect were used as phenotypic values of a tree in subsequent GWAS and genomic prediction modelling. The MLM was implemented in the “lmer” function of the R package lme4 ver. 1.1–7^57^. Phenotypic variations of the nine traits evaluated in both parental and breeding populations were visualized as jitter plots superimposed onto boxplots by using the R packages ggplot2 ver. 1.0.1^58^ and Rmisc ver. 1.5^59^.

### SNP genotyping data

Genomic DNA was extracted according to Yamamoto *et al*.^29^. We genotyped 1,536 SNPs in the parental population and seven families of the breeding population (502, 506, 510, 518, 520, and 523), and 768 SNPs in the remaining nine families. SNPs were genotyped by using a custom-designed SNP array for Illumina GoldenGate Genotyping Assay (Illumina Inc.) (Supplementary Methods; Supplementary Data S1). Each SNP genotype was converted to 1 (AA homozygotes), −1 (BB homozygotes), or 0 (AB heterozygotes). The sporadic missing genotypes in the parental population and the seven families were imputed using the R package missForest ver. 1.4^60^. The 768 SNPs of the remaining nine F_1_ families were extended to the 1,536 SNPs using the same imputation method. Finally, markers that were not polymorphic were removed and a total of 1,506 SNPs for the parental and breeding populations were obtained.

### Linkage disequilibrium estimation and population structure analysis

Squared correlation coefficients (*r*^2^) between pairs of 563 SNPs that were mapped on the genetic linkage map (see Supplementary Methods; Supplementary Fig. S1) were calculated and plotted against map distance (cM) between the corresponding markers within the same LG. To model the relationship between the *r*^2^ values and linkage map distances, local polynomial regression with kernel weight was conducted using the “locpoly” function in the R package KernSmooth ver. 2.23–13^61^. Linkage map distances between adjacent markers were 0–26.14 cM (mean, 2.56 cM). The *r*^2^ values between pairs of unlinked markers were also calculated.

The genetic structure in the parental population was estimated using hierarchical clustering and PCA. Hierarchical clustering based on Ward’s method^62^ with Euclidean distance and PCA were conducted using the R functions “hclust” and “prcomp”, respectively. The principal component (PC) scores of the breeding populations were calculated based on the eigen vectors obtained in the PCA of the parental population to locate the breeding populations in the PCA space of the parental population.

### GWAS

Single-locus GWAS was conducted using an MLM^63^ implemented in the “GWA” function of the R package rrBLUP ver. 4.0^64^. To avoid spurious associations due to population structure, a kinship matrix and the scores of the first four PCs were included in the MLM as random and fixed effects, respectively. The kinship matrix was computed using the “A.mat” function of the R package rrBLUP. The optimal number of PCs was determined by estimating the variances of PC scores. The variance of PC score decreased rapidly until PC4 and only gradually thereafter (Supplementary Fig. S13). FDR was calculated for all the traits evaluated in this study using the modified “GWAS” function of the R package rrBLUP ver. 4.3^64^. Multi-locus GWAS methods, which are more suitable for complex traits controlled by multiple loci and show high detection power under less stringent criteria than the single-locus GWAS, have recently been proposed^44^. Three multi-locus GWASs (FASTmrEMMA^65^, ISIS EM-BLASSO^66^, and mrMLM^44^) were also conducted using the R package mrMLM ver. 3.0^44^. The kinship matrix used in the single-locus GWAS was also used in the three multi-locus GWAS methods. The significant associated SNPs were determined by the critical threshold of LOD score ≥3 as described in Tamba *et al*.^66^, Wang *et al*.^44^, and Wen *et al*.^65^.

### Single-trait genomic prediction

To evaluate the accuracy of genomic prediction on a single-trait basis, we used 12 methods: genomic best linear unbiased prediction (GBLUP) with ridge kernel regression (RR) or Gaussian kernel regression (GAUSS), Random Forest, Ridge Regression, Lasso, Elastic Net, Bayesian Ridge Regression, Bayesian Lasso, BayesA, BayesB, BayesC, and the mean prediction of all the above methods. Prediction models based on the 12 methods were built as described by Minamikawa *et al*.^25^. The prediction accuracy of the models was cross-validated as described below.

To evaluate the potential of MAS based on SNPs detected in GWAS with the parental or combined population without genotypes targeted by genomic prediction, the top one, two, or three peak SNPs with high −log_10_(*p*) values for each trait were entered in single-trait-targeted MLR of the R function “lm”^67,68^ unless the squared correlation coefficient between the SNPs was ≥0.6 (to prevent multicollinearity). In general, one or few markers are used for MAS to improve traits controlled by a small number of major genes and/or large QTLs. The prediction accuracies of the models were compared to that of GBLUP (RR), which treats all SNPs.

To evaluate the importance of dominance effects on genomic prediction, a multi-kernel model (considering both additive and dominance effects) was compared with a single-kernel model (considering only additive effects), as described by Minamikawa *et al*.^25^. Both are single-trait models and are implemented in the R package of BGLR ver. 1.0.3^69^. The additive (${\sigma }_{a}^{2}$) and dominance (${\sigma }_{d}^{2}$) genetic variances and residual variance (${\sigma }_{e}^{2}$) of the parental and combined populations were estimated with the multi-kernel model. Narrow-sense heritability (*h*^2^) of each trait was computed as the ratio of ${\sigma }_{a}^{2}$ to the total phenotypic variance (${\sigma }_{a}^{2}+{\sigma }_{d}^{2}+{\sigma }_{e}^{2}$). That is, ${h}^{2}={\sigma }_{a}^{2}/({\sigma }_{a}^{2}+{\sigma }_{d}^{2}+{\sigma }_{e}^{2})$.

### Multi-trait genomic prediction

To evaluate the accuracy of genomic prediction on a multi-trait basis, we employed four methods, which took genetic correlations among traits into account, and compared their accuracy with that of the methods based on a single trait. A Bayesian multivariate mixed model fitted via variational Bayes named PHENIX was tested by using the R package phenix ver. 1.0^70^. We also used the R package glmnet ver. 2.0–10 for three different linear regression-based multi-response Gaussian family methods: Ridge Regression (alpha = 0), Lasso (alpha = 1), and Elastic Net (alpha = 0.5)^71^.

### Cross-validation of genomic prediction accuracy

To evaluate the accuracy of genomic prediction for a family in the breeding population, we conducted five different types of validation: (i) training with the parental population, (ii) training with the breeding populations excluding the target family, (iii) training with the combined population (parental and breeding populations) excluding the target family, (iv) 10-fold cross-validation (CV) with the target family (only for family 540; leave-one-out CV was performed because of a few F_1_ genotypes), and (v) 10-fold CV with the combined data from (iii) and (iv). The CVs in types (iv) and (v) were repeated 3 times, and the identical pattern of folds (i.e., random separation of samples into 10 folds) was adapted to all prediction models in each CV. The 10-fold CV repeated 5 times was also conducted to evaluate the accuracy of genomic prediction in the parental population, because nine traits were evaluated only in that population (Table 1). Generally, phenotypic information of the family targeted by GS is not available in actual breeding programs. If some elite cultivars are obtained from one family, more F_1_ genotypes from the family will be evaluated to obtain the better F_1_ genotypes, and the phenotypic information of the family will be available for the construction of genomic prediction model. The prediction accuracy was evaluated with the Pearson’s correlation coefficient (*r*) between observed and predicted genotypic values. When estimated *r* was less than 0, it was regarded as 0. The prediction accuracy for each family and for the combination of all families was calculated. Root-mean squared errors between the observed and predicted values were also calculated for comparing the prediction accuracy among the families, because the magnitude of segregated variation differed considerably among the families (Supplementary Fig. S10). When the variation in a family is small, correlation in the family might be low even though the prediction accuracy is high.

## Electronic supplementary material

Supplementary information

Supplementary data
