# Supplementary material for: Genome-wide association study and genomic prediction using parental and breeding populations of Japanese pear (Pyrus pyrifolia Nakai)
Source: Sci Rep. 2018 Aug 10;8:11994. doi: 10.1038/s41598-018-30154-w (PMC6086889; doi:10.1038/s41598-018-30154-w)
Supplement: Supplementary file 1 — Supplementary information [file 41598_2018_30154_MOESM1_ESM.pdf]

Supplementary information

Genome-wide association study and genomic prediction using parental and breeding populations of Japanese pear (*Pyrus pyrifolia* Nakai)

Mai F. Minamikawa[1], Norio Takada[2], Shingo Terakami[2], Toshihiro Saito[2], Akio Onogi[1],
Hiromi Kajiya-Kanegae[1], Takeshi Hayashi[3], Toshiya Yamamoto[2], Hiroyoshi Iwata[1*]

[1]Laboratory of Biometry and Bioinformatics, Department of Agricultural and Environmental Biology, Graduate School of Agricultural and Life Sciences, The University of Tokyo, 1-1-1 Yayoi, Bunkyo, Tokyo 113-8657, Japan
[2]Institute of Fruit Tree and Tea Science, National Agriculture and Food Research Organization (NARO), 2-1 Fujimoto, Tsukuba, Ibaraki 305-8605, Japan
[3]Institute of Crop Science, NARO, 2-1-2 Kannondai, Tsukuba, Ibaraki 305-8518, Japan

*To whom correspondence should be addressed: E-mail: aiwata@mail.ecc.u-tokyo.ac.jp

Supplementary Tables S1-S9
Supplementary Figures S1-S13
Supplementary Methods
References

Supplementary Table S1. Parental pear population used in this study.

Vatiety No.	Variety	Type	Release year	Vatiety No.	Variety	Type	Release year
1	Akemizu	Modern	1997	44	Natsuhikari	Modern	1995
2	Akiakari	Modern	2003*	45	Natsushizuku	Modern	2008*
3	Akibae	Modern	1997	46	Niitaka	Old	1927
4	Akizuki	Modern	2001*	47	Nijisseiki	Indigenous	
5	Amanogawa	Indigenous		48	Nikkori	Modern	1996
6	Asahi	Old		49	O9	Breeding line	
7	Atago	Old	1927	50	Okusankichi	Indigenous	
8	C2	Breeding line		51	Oushuu	Modern	2003*
9	Chikusui	Modern	1989*	52	Ri14	Breeding line	
10	Chojuro	Indigenous		53	Rinka	Modern	2015*
11	Chouju	Modern	1973	54	Sagami	Old	1927
12	Cili	Indigenous		55	Seigyoku	Old	1952
13	Doitsu	Indigenous		56	Seiryuu	Indigenous	
14	Gion	Old	1927	57	Shinchuu	Indigenous	
15	Hakkou	Modern	1972*	58	Shinkou	Old	1941
16	Hatsuaki	Old		59	Shinsei	Modern	1984*
17	Hatsumaru	Modern	2015*	60	Shinseiki	Old	1945
18	Hattatsu	Old	1940	61	Shinsetsu	Modern	1949
19	Hayatama	Modern	1968*	62	Shinsui	Modern	1965*
20	Heiwa	Old		63	Shuugyoku	Modern	1988*
21	Higashino	Old		64	Shuurei	Modern	2003*
22	Hiratsuka16	Modern		65	Suisei	Modern	1955*
23	Hokushin	Modern	1997	66	Taihaku	Indigenous	
24	Hoshiakari	Modern	2015*	67	Tama	Modern	1971
25	Hosui	Modern	1972*	68	Tsukuba43	Breeding line	
26	Hougetsu	Modern	1994*	69	Tsukuba52	Breeding line	
27	I33	Breeding line		70	Tsukuba53	Breeding line	
28	Ichiharawase	Indigenous		71	Tsukuba57	Breeding line	
29	Imamuraaki	Indigenous		72	Wakahikari	Modern	1992
30	Inagi	Old		73	Waseaka	Indigenous	
31	Ishiiwase	Old	1921	74	Wasekouzou	Indigenous	
32	Kanta	Modern	2015*	75	Yachiyo	Old	
33	Kikusui	Old	1927	76	Yali	Indigenous	
34	Kimitsukawase	Old	1940	77	Yanaga	Old	1935
35	Kinchaku	Indigenous		78	Yasato	Modern	1990*
36	Kisui	Modern	1990	79	162-29	Breeding line	
37	Kosui	Modern	1959*	80	266-27	Breeding line	
38	Kougetsu	Old		81	373-55	Breeding line	
39	Kumoi	Modern	1955*	82	42-6	Breeding line	
40	Kunitomi	Indigenous		83	450-63	Breeding line	
41	Meigetsu	Indigenous		84	450-7	Breeding line	
42	Mishirazu	Indigenous		85	48-96	Breeding line	
43	Nangetsu	Modern	1997	86	92-7	Breeding line	

*Modern elite cultivars bred by NARO Institute of Fruit Tree Science (Ibaraki, Japan) after release years.

'Cili' and 'Yali' are Chinese pear and others are Japanese pear.

Supplementary Table S2. Breeding pear population used in this study.

Family No.	Female	Vatiety No.	Male	Vatiety No.	Number of genotypes
502	Tsukuba52	69	Oushuu	51	30
506	Akizuki	4	Chikusui	9	40
510	Shuurei	64	Hoshiakari	24	90
518	Yasato	78	Kanta	32	34
520	Oushuu	51	Shinsui	62	26
521	Kosui	37	Kanta	32	26
523	Akizuki	4	373-55	81	123
538	Akizuki	4	Hoshiakari	24	30
539	Akizuki	4	450-63	83	62
540	Rinka	53	Hoshiakari	24	6
541	Kosui	37	Hoshiakari	24	49
542	Akiakari	2	450-7	84	33
543	Natsushizuku	45	Hoshiakari	24	83
545	Kosui	37	Inagi	30	33
546	Akizuki	4	Akiakari	2	27
547	Akiakari	2	Okuroku*	-	73

*Varietiy not included in the parental population (Supplementary Table S1).

Supplementary Table S3. Significant SNPs detected by single-locus GWAS using the parental population (Supplementary Figure S3).

Trait	SNP ID	Linkage Group	Position (cM)	$-\log_{10}(p)$ value	Minor allele frequency (MAF)
Aci	scaffold361.0_21343	18	−	4.636	0.256
Aci	scaffold361.0_273919	18	−	4.478	0.250
FruC	scaffold49.2_289494	8	9.38	11.192	0.448
FruD	scaffold408.0_132801	18	−	4.929	0.494
BSR1	scaffold153.0_587903	18	−	4.288	0.238
BSR1	scaffold181.0_182602	18	−	5.122	0.174
BSR2	scaffold153.0_587903	18	−	5.994	0.238
BSR2	scaffold181.0_182602	18	−	6.634	0.174

Supplementary Table S4. Significant SNPs detected by single-locus GWAS using the combined population (Figure 3).

Trait	SNP ID	Linkage Group	Position (cM)	$-\log_{10}(p)$ value	Minor allele frequency (MAF)
HarT	scaffold898.0_23949	3	56.99	4.285	0.458
HarT	scaffold24.0_1139851	10	73.45	4.228	0.172
HarT	scaffold23.0_1195054	15	60.00	4.525	0.214
HarT	scaffold316.0_64431	18	−	8.321	0.095
HarT	scaffold717.0_1897	18	−	8.203	0.101
HarT	scaffold1220.0_42454	18	−	3.883	0.476
FruW	scaffold32.0_452809	10	55.37	4.347	0.474
FruW	scaffold24.0_1139851	10	73.45	5.169	0.172
FruW	scaffold525.0_282259	18	−	4.643	0.039
Aci	scaffold540.0_9871	6	44.38	3.687	0.129
Aci	scaffold1356.0_31412	6	45.52	5.540	0.351
Aci	scaffold67.0_299716	6	50.91	4.858	0.304
Aci	scaffold19.0_974379	18	−	3.723	0.038
Aci	scaffold21.0_41995	18	−	3.748	0.136
Aci	scaffold201.0_477821	18	−	4.061	0.032
Aci	scaffold389.0_202556	18	−	4.957	0.140
Aci	scaffold729.0_72113	18	−	4.638	0.135
Aci	scaffold908.0_16975	18	−	6.126	0.156
FruC	scaffold131.0_429923	8	3.74	3.935	0.424
FruC	scaffold131.0_744834	8	5.79	5.216	0.159
FruC	scaffold288.1_42614	8	7.22	11.390	0.152
FruC	scaffold288.1_60712	8	7.57	7.888	0.148
FruC	scaffold49.2_289494	8	9.38	37.473	0.478
FruC	scaffold49.1_715919	8	10.49	4.190	0.305
FruD	scaffold49.2_289494	8	9.38	7.043	0.478
HeaR	scaffold24.0_1139851	10	73.45	5.064	0.172

Supplementary Table S5. Significant SNPs detected by multi-locus GWAS using the parental population.

Trait	Method	SNP ID	Linkage Group	Position (cM)	LOD score	Minor allele frequency (MAF)
HarT	FASTmrEMMA	scaffold808.0_173655	18	−	5.764	0.186
HarT	FASTmrEMMA	scaffold876.0_10416	18	−	3.001	0.378
HarT	ISIS EM-BLASSO	scaffold1231.0_82832	3	77.35	4.648	0.477
HarT	ISIS EM-BLASSO	scaffold23.0_1195054	15	60.00	6.724	0.320
HarT	ISIS EM-BLASSO	scaffold3.0_2040751	18	−	3.564	0.099
HarT	ISIS EM-BLASSO	scaffold757.0_26308	18	−	4.903	0.244
HarT	mrMLM	scaffold23.0_1195054	15	60.00	5.666	0.327
HarT	mrMLM	scaffold3.0_2040751	18	−	5.908	0.101
FruW	FASTmrEMMA	scaffold1239.0_16321	1	23.08	3.649	0.221
FruW	FASTmrEMMA	scaffold47.0_53121	6	82.81	5.277	0.145
FruW	FASTmrEMMA	scaffold131.0_744834	8	5.79	5.362	0.157
FruW	ISIS EM-BLASSO	scaffold1239.0_16321	1	23.08	4.809	0.221
FruW	ISIS EM-BLASSO	scaffold20.0_999886	1	66.49	3.367	0.395
FruW	ISIS EM-BLASSO	scaffold47.0_53121	6	82.81	5.174	0.145
FruW	ISIS EM-BLASSO	scaffold131.0_744834	8	5.79	3.467	0.157
FruH	FASTmrEMMA	scaffold1068.0_63043	18	−	5.279	0.071
FruH	FASTmrEMMA	scaffold1111.0_13428	18	−	4.499	0.265
FruH	ISIS EM-BLASSO	scaffold728.0_2190	18	−	3.228	0.494
FruH	ISIS EM-BLASSO	scaffold942.0_71906	18	−	4.665	0.047
FruH	ISIS EM-BLASSO	scaffold1111.0_13428	18	−	3.680	0.265
FruH	ISIS EM-BLASSO	scaffold1537.0_22947	18	−	4.813	0.341
FruH	mrMLM	scaffold1050.0_2425	5	79.54	4.857	0.259
FruH	mrMLM	scaffold1150.0_66019	7	39.65	6.325	0.235
FruH	mrMLM	scaffold857.0_157654	13	60.28	3.008	0.145
FruH	mrMLM	scaffold1227.0_4835	17	88.12	4.191	0.331
SugC	mrMLM	scaffold1494.0_12388	1	1.31	3.165	0.386
Aci	FASTmrEMMA	scaffold249.0_47812	12	22.89	3.756	0.300
Aci	FASTmrEMMA	scaffold1121.0_58127	18	−	4.540	0.135
Aci	ISIS EM-BLASSO	scaffold249.0_47812	12	22.89	4.225	0.300
Aci	ISIS EM-BLASSO	scaffold1209.0_74507	13	5.32	3.236	0.341
Aci	ISIS EM-BLASSO	scaffold514.0_228066	18	−	5.218	0.106
Aci	mrMLM	scaffold357.0_9771	2	15.10	4.424	0.024
Aci	mrMLM	scaffold424.0_302546	3	76.38	7.238	0.157
Aci	mrMLM	scaffold249.0_47812	12	22.89	6.286	0.295
Aci	mrMLM	scaffold1209.0_74507	13	5.32	3.173	0.331
FruC	FASTmrEMMA	scaffold49.2_289494	8	9.38	12.913	0.452
FruC	ISIS EM-BLASSO	scaffold49.2_289494	8	9.38	23.321	0.452
FruC	ISIS EM-BLASSO	scaffold1136.0_77338	18	−	4.608	0.304
FruD	ISIS EM-BLASSO	scaffold110.0_587351	5	20.71	4.235	0.204
FruD	ISIS EM-BLASSO	scaffold408.0_132801	18	−	4.345	0.494
HeaR	FASTmrEMMA	scaffold334.0_81046	18	−	3.368	0.394
HeaR	ISIS EM-BLASSO	scaffold418.0_1182	18	−	4.226	0.065
HeaR	ISIS EM-BLASSO	scaffold422.0_49934	18	−	3.138	0.094
HeaR	ISIS EM-BLASSO	scaffold1211.0_34964	18	−	3.189	0.365
HeaR	mrMLM	scaffold982.0_129342	3	27.23	4.059	0.187
HeaR	mrMLM	scaffold1520.0_25969	8	21.88	3.569	0.090
FruS	ISIS EM-BLASSO	scaffold152.0_554461	18	−	5.664	0.299
FruS	ISIS EM-BLASSO	scaffold892.0_73114	18	−	3.840	0.451
FruS	ISIS EM-BLASSO	scaffold1220.0_42454	18	−	3.169	0.396
Rust	FASTmrEMMA	scaffold49.2_289494	8	9.38	4.111	0.450
Rust	mrMLM	scaffold638.0_15107	1	2.84	3.370	0.160
Rust	mrMLM	scaffold782.0_75520	3	69.63	3.160	0.115
Appear	ISIS EM-BLASSO	scaffold128.0_267730	12	10.00	3.386	0.276
Groove	ISIS EM-BLASSO	scaffold117.0_695278	1	3.43	5.531	0.373
Groove	ISIS EM-BLASSO	scaffold1225.0_28264	14	23.77	3.907	0.329
Groove	ISIS EM-BLASSO	scaffold786.0_20436	17	95.47	5.485	0.411
Groove	ISIS EM-BLASSO	scaffold955.0_33154	18	−	3.425	0.354
Groove	mrMLM	scaffold1225.0_28264	14	23.77	4.979	0.325
Groove	mrMLM	scaffold72.0_134148	18	−	4.411	0.026
BSR1	FASTmrEMMA	scaffold181.0_182602	18	−	6.284	0.173
BSR1	ISIS EM-BLASSO	scaffold181.0_182602	18	−	7.060	0.173
BSR1	ISIS EM-BLASSO	scaffold408.0_132801	18	−	3.737	0.494
BSR1	ISIS EM-BLASSO	scaffold637.0_4413	18	−	3.463	0.173
BSR2	FASTmrEMMA	scaffold181.0_182602	18	−	3.913	0.173
BSR2	ISIS EM-BLASSO	scaffold181.0_182602	18	−	4.527	0.173
BSR2	ISIS EM-BLASSO	scaffold1679.0_4401	18	−	3.148	0.048
TreV	ISIS EM-BLASSO	scaffold387.0_112168	4	35.92	3.826	0.399
SpuN	ISIS EM-BLASSO	scaffold7.0_28955	3	32.00	3.789	0.256
SpuN	ISIS EM-BLASSO	scaffold83.0_101251	13	12.68	3.761	0.482
SpuN	mrMLM	scaffold341.0_456949	1	11.09	3.333	0.195
SpuN	mrMLM	scaffold305.0_186986	2	55.58	3.171	0.165

Supplementary Table S6. Significant SNPs detected by multi-locus GWAS using the combined population.

Trait	Method	SNP ID	Linkage Group	Position (cM)	LOD score	Minor allele frequency (MAF)
HarT	FASTmrEMMA	scaffold898.0_23949	3	56.99	3.219	0.458
HarT	FASTmrEMMA	scaffold24.0_1139851	10	73.45	15.255	0.172
HarT	FASTmrEMMA	scaffold572.0_123960	15	52.37	3.011	0.217
HarT	FASTmrEMMA	scaffold23.0_1195054	15	60.00	10.798	0.215
HarT	FASTmrEMMA	scaffold717.0_1897	18	−	45.167	0.101
HarT	FASTmrEMMA	scaffold930.0_4168	18	−	20.402	0.062
HarT	FASTmrEMMA	scaffold1410.0_2431	18	−	16.856	0.497
HarT	ISIS EM-BLASSO	scaffold275.0_83851	1	33.55	8.695	0.462
HarT	ISIS EM-BLASSO	scaffold20.0_999886	1	66.49	4.352	0.390
HarT	ISIS EM-BLASSO	scaffold183.0_380750	2	12.57	3.911	0.445
HarT	ISIS EM-BLASSO	scaffold2.0_2254158	3	10.53	3.838	0.290
HarT	ISIS EM-BLASSO	scaffold1231.0_82832	3	77.35	8.647	0.430
HarT	ISIS EM-BLASSO	scaffold1221.0_38819	5	0.50	3.320	0.357
HarT	ISIS EM-BLASSO	scaffold1028.0_6815	7	58.58	4.658	0.484
HarT	ISIS EM-BLASSO	scaffold131.0_744834	8	5.79	6.636	0.159
HarT	ISIS EM-BLASSO	scaffold24.0_1139851	10	73.45	7.947	0.172
HarT	ISIS EM-BLASSO	scaffold1159.0_72222	11	48.33	11.867	0.389
HarT	ISIS EM-BLASSO	scaffold572.0_123960	15	52.37	5.315	0.217
HarT	ISIS EM-BLASSO	scaffold23.0_1195054	15	60.00	15.027	0.215
HarT	ISIS EM-BLASSO	scaffold46.0_851647	18	−	3.715	0.083
HarT	ISIS EM-BLASSO	scaffold50.0_149665	18	−	4.334	0.120
HarT	ISIS EM-BLASSO	scaffold174.0_185983	18	−	15.329	0.351
HarT	ISIS EM-BLASSO	scaffold194.0_394721	18	−	4.407	0.395
HarT	ISIS EM-BLASSO	scaffold316.0_64431	18	−	20.473	0.095
HarT	ISIS EM-BLASSO	scaffold486.0_254268	18	−	3.141	0.434
HarT	ISIS EM-BLASSO	scaffold490.0_259677	18	−	4.266	0.118
HarT	ISIS EM-BLASSO	scaffold686.0_15414	18	−	8.050	0.082
HarT	ISIS EM-BLASSO	scaffold828.0_14398	18	−	6.816	0.186
HarT	ISIS EM-BLASSO	scaffold1220.0_42454	18	−	17.307	0.476
HarT	ISIS EM-BLASSO	scaffold1226.0_61995	18	−	3.489	0.254
HarT	ISIS EM-BLASSO	scaffold1385.0_28347	18	−	7.367	0.113
FruW	FASTmrEMMA	scaffold24.0_1139851	10	73.45	16.333	0.172
FruW	FASTmrEMMA	scaffold382.0_38383	18	−	7.570	0.485
FruW	FASTmrEMMA	scaffold468.0_3696	18	−	5.159	0.054
FruW	ISIS EM-BLASSO	scaffold275.0_83851	1	33.55	5.868	0.462
FruW	ISIS EM-BLASSO	scaffold330.0_111785	1	43.86	3.065	0.488
FruW	ISIS EM-BLASSO	scaffold20.0_999886	1	66.49	3.168	0.390
FruW	ISIS EM-BLASSO	scaffold1201.0_66861	3	28.92	5.449	0.289
FruW	ISIS EM-BLASSO	scaffold37.0_696113	7	0.00	3.824	0.163
FruW	ISIS EM-BLASSO	scaffold276.0_126008	7	34.18	4.154	0.438
FruW	ISIS EM-BLASSO	scaffold1665.0_11891	8	23.72	5.789	0.268
FruW	ISIS EM-BLASSO	scaffold32.0_452809	10	55.37	3.655	0.474
FruW	ISIS EM-BLASSO	scaffold24.0_1139851	10	73.45	13.836	0.172
FruW	ISIS EM-BLASSO	scaffold472.0_28532	12	53.51	8.941	0.199
FruW	ISIS EM-BLASSO	scaffold83.0_101251	13	12.68	4.817	0.324
FruW	ISIS EM-BLASSO	scaffold819.0_38650	13	71.59	3.591	0.248
FruW	ISIS EM-BLASSO	scaffold1167.0_29739	16	33.78	4.684	0.084
FruW	ISIS EM-BLASSO	scaffold137.0_502463	17	39.41	3.520	0.271
FruW	ISIS EM-BLASSO	scaffold169.0_265760	18	−	3.671	0.347
FruW	ISIS EM-BLASSO	scaffold174.0_185983	18	−	3.301	0.351
FruW	ISIS EM-BLASSO	scaffold226.0_62840	18	−	3.265	0.151
FruW	ISIS EM-BLASSO	scaffold382.0_38383	18	−	3.100	0.485
FruW	ISIS EM-BLASSO	scaffold428.0_32484	18	−	3.646	0.340
FruW	ISIS EM-BLASSO	scaffold480.0_41660	18	−	5.932	0.115
FruW	ISIS EM-BLASSO	scaffold613.0_214594	18	−	3.343	0.113
FruW	ISIS EM-BLASSO	scaffold863.0_3099	18	−	10.396	0.153
FruW	mrMLM	scaffold583.0_7194	1	17.40	3.023	0.087
FruW	mrMLM	scaffold94.0_501159	2	8.97	4.729	0.306
FruW	mrMLM	scaffold110.0_587351	5	20.71	3.639	0.190
FruW	mrMLM	scaffold37.0_696113	7	0.00	3.047	0.164
FruW	mrMLM	scaffold1520.0_25969	8	21.88	5.671	0.249
FruW	mrMLM	scaffold24.0_1139851	10	73.45	12.606	0.172
FruW	mrMLM	scaffold242.0_168968	12	59.19	6.557	0.173
FruW	mrMLM	scaffold3.0_2040751	18	−	15.162	0.063
FruH	FASTmrEMMA	scaffold1150.0_66019	7	39.65	10.057	0.140
FruH	FASTmrEMMA	scaffold47.0_520566	18	−	18.470	0.041
FruH	FASTmrEMMA	scaffold409.0_40479	18	−	7.225	0.059
FruH	FASTmrEMMA	scaffold611.0_28036	18	−	23.740	0.033
FruH	FASTmrEMMA	scaffold918.0_71319	18	−	7.943	0.070
FruH	FASTmrEMMA	scaffold1170.0_25556	18	−	4.520	0.002
FruH	mrMLM	scaffold357.0_9771	2	15.10	4.122	0.161
FruH	mrMLM	scaffold63.0_567705	3	0.79	18.074	0.426
FruH	mrMLM	scaffold1150.0_66019	7	39.65	14.509	0.140
FruH	mrMLM	scaffold192.0_30648	10	45.85	6.193	0.306
FruH	mrMLM	scaffold309.0_97597	12	32.70	14.580	0.271
FruH	mrMLM	scaffold374.0_46624	14	29.13	4.442	0.102

Supplementary Table S6. *(Continued)*

Trait	Method	SNP ID	Linkage Group	Position (cM)	LOD score	Minor allele frequency (MAF)
SugC	FASTmrEMMA	scaffold1114.0_9449	8	49.35	3.450	0.451
SugC	ISIS EM-BLASSO	scaffold40.0_716843	5	27.56	6.204	0.245
SugC	ISIS EM-BLASSO	scaffold949.0_17680	7	57.76	6.224	0.475
SugC	ISIS EM-BLASSO	scaffold994.0_47472	7	68.68	5.484	0.365
SugC	ISIS EM-BLASSO	scaffold794.0_46450	17	3.71	3.937	0.334
SugC	ISIS EM-BLASSO	scaffold51.0_241255	18	−	5.520	0.080
SugC	ISIS EM-BLASSO	scaffold106.0_54398	18	−	6.051	0.275
SugC	ISIS EM-BLASSO	scaffold260.0_139237	18	−	7.655	0.277
SugC	ISIS EM-BLASSO	scaffold288.2_10209	18	−	4.751	0.163
SugC	mrMLM	scaffold890.0_79775	2	18.09	3.725	0.228
SugC	mrMLM	scaffold297.0_19047	4	7.34	3.155	0.281
SugC	mrMLM	scaffold1.0_3362771	5	0.14	3.608	0.134
SugC	mrMLM	scaffold712.0_66900	7	5.95	13.329	0.159
SugC	mrMLM	scaffold288.1_42614	8	7.22	3.842	0.152
SugC	mrMLM	scaffold622.0_137668	11	0.62	4.026	0.153
Aci	FASTmrEMMA	scaffold1356.0_31412	6	45.52	4.668	0.351
Aci	FASTmrEMMA	scaffold19.0_974379	18	−	7.842	0.038
Aci	FASTmrEMMA	scaffold201.0_477821	18	−	10.297	0.031
Aci	FASTmrEMMA	scaffold374.0_30760	18	−	9.693	0.137
Aci	FASTmrEMMA	scaffold908.0_16975	18	−	4.250	0.156
Aci	FASTmrEMMA	scaffold1121.0_58127	18	−	10.099	0.014
Aci	ISIS EM-BLASSO	scaffold330.0_111785	1	43.86	13.347	0.488
Aci	ISIS EM-BLASSO	scaffold333.0_110956	4	29.18	10.294	0.456
Aci	ISIS EM-BLASSO	scaffold1356.0_31412	6	45.52	17.665	0.351
Aci	ISIS EM-BLASSO	scaffold760.0_52180	6	58.83	3.190	0.405
Aci	ISIS EM-BLASSO	scaffold87.0_30443	7	93.87	6.821	0.343
Aci	ISIS EM-BLASSO	scaffold398.0_27591	8	9.75	3.062	0.237
Aci	ISIS EM-BLASSO	scaffold57.0_993818	10	87.60	4.160	0.440
Aci	ISIS EM-BLASSO	scaffold48.0_568154	13	5.92	4.948	0.314
Aci	ISIS EM-BLASSO	scaffold1225.0_28264	14	23.77	3.519	0.412
Aci	ISIS EM-BLASSO	scaffold23.0_1195054	15	60.00	3.509	0.215
Aci	ISIS EM-BLASSO	scaffold19.0_13364	18	−	3.515	0.468
Aci	ISIS EM-BLASSO	scaffold81.0_644865	18	−	3.628	0.165
Aci	ISIS EM-BLASSO	scaffold133.0_17487	18	−	4.052	0.080
Aci	ISIS EM-BLASSO	scaffold194.0_394721	18	−	3.921	0.396
Aci	ISIS EM-BLASSO	scaffold201.0_477821	18	−	7.253	0.031
Aci	ISIS EM-BLASSO	scaffold374.0_30760	18	−	5.764	0.137
Aci	ISIS EM-BLASSO	scaffold771.0_9327	18	−	3.341	0.050
Aci	ISIS EM-BLASSO	scaffold964.0_77711	18	−	3.791	0.462
Aci	ISIS EM-BLASSO	scaffold1495.0_1060	18	−	3.090	0.393
FruC	FASTmrEMMA	scaffold131.0_429923	8	3.74	10.445	0.424
FruC	FASTmrEMMA	scaffold131.0_744834	8	5.79	5.363	0.158
FruC	FASTmrEMMA	scaffold288.1_60712	8	7.57	5.334	0.148
FruC	FASTmrEMMA	scaffold49.2_289494	8	9.38	61.094	0.478
FruC	mrMLM	scaffold797.0_108491	1	46.65	5.781	0.106
FruC	mrMLM	scaffold209.0_216767	2	0.00	10.635	0.391
FruC	mrMLM	scaffold131.0_744834	8	5.79	35.017	0.157
FruD	FASTmrEMMA	scaffold49.2_289494	8	9.38	12.824	0.478
FruD	FASTmrEMMA	scaffold258.0_373122	18	−	3.017	0.069
FruD	ISIS EM-BLASSO	scaffold49.2_289494	8	9.38	13.780	0.478
FruD	mrMLM	scaffold23.0_1195054	15	60.00	3.959	0.215
HeaR	FASTmrEMMA	scaffold24.0_1139851	10	73.45	7.243	0.172
HeaR	FASTmrEMMA	scaffold286.0_144171	11	8.06	5.078	0.452
HeaR	FASTmrEMMA	scaffold1276.0_15622	18	−	12.570	0.053
HeaR	ISIS EM-BLASSO	scaffold24.0_1139851	10	73.45	8.267	0.172
HeaR	mrMLM	scaffold1649.0_1278	3	75.02	3.665	0.300
HeaR	mrMLM	scaffold373.0_409875	6	63.78	4.278	0.302
HeaR	mrMLM	scaffold24.0_1139851	10	73.45	7.423	0.172
HeaR	mrMLM	scaffold376.0_144978	15	102.69	12.056	0.168
HeaR	mrMLM	scaffold214.0_7849	17	3.85	3.896	0.299
WatC	ISIS EM-BLASSO	scaffold98.0_498361	3	9.49	5.653	0.333
WatC	ISIS EM-BLASSO	scaffold286.0_144171	11	8.06	6.358	0.452
WatC	ISIS EM-BLASSO	scaffold914.0_45652	13	44.05	3.297	0.330
WatC	ISIS EM-BLASSO	scaffold76.0_497313	15	64.59	4.335	0.279
WatC	ISIS EM-BLASSO	scaffold699.0_116846	18	−	5.376	0.231
WatC	mrMLM	scaffold98.0_498361	3	9.49	3.581	0.333
WatC	mrMLM	scaffold619.0_3807	10	50.82	3.246	0.180
WatC	mrMLM	scaffold622.0_137668	11	0.62	7.286	0.153
WatC	mrMLM	scaffold698.0_17076	12	4.15	3.767	0.088

Supplementary Table S7. Phenotypic correlation in the parental population.

	HarT	FruW	FruH	SugC	Aci	FruC	FruD	HeaR	WatC	SWatC	FruS	Rust	Appear	Groove	BSR1	BSR2	TreV	SpuN
HarT	1.00	0.65	-0.07	-0.03	-0.53	0.04	0.15	0.02	-0.11	-0.07	0.06	-0.08	-0.21	-0.22	0.21	0.23	0.14	0.33
FruW		1.00	-0.13	0.03	-0.24	0.19	0.09	-0.19	-0.18	-0.04	-0.12	-0.16	-0.03	0.01	0.25	0.26	0.20	0.28
FruH			1.00	-0.22	-0.42	-0.05	-0.04	0.09	-0.19	-0.15	0.02	-0.08	0.14	-0.23	-0.26	-0.30	-0.10	-0.11
SugC				1.00	0.10	0.03	0.09	0.06	-0.12	0.02	-0.04	0.20	-0.06	0.04	0.09	0.08	-0.02	0.10
Aci					1.00	0.03	0.01	-0.17	0.08	0.07	-0.07	0.08	0.02	0.23	-0.01	-0.04	-0.04	-0.24
FruC						1.00	-0.42	0.14	0.00	0.07	-0.01	-0.67	0.03	0.28	-0.10	-0.13	-0.02	0.26
FruD							1.00	-0.02	0.00	0.13	0.15	0.22	0.15	-0.21	-0.12	-0.08	-0.17	-0.21
HeaR								1.00	0.19	0.14	-0.14	-0.09	0.01	-0.09	0.02	-0.02	-0.10	0.16
WatC									1.00	0.70	-0.12	-0.03	0.09	-0.04	-0.04	0.00	-0.02	0.14
SWatC										1.00	0.06	-0.03	0.05	-0.09	0.06	0.04	-0.10	0.01
FruS											1.00	0.03	-0.20	0.01	-0.11	-0.13	-0.08	-0.27
Rust												1.00	-0.27	-0.12	0.14	0.17	0.07	-0.13
Appear													1.00	-0.40	-0.04	-0.01	-0.08	0.07
Groove														1.00	-0.02	-0.05	0.15	-0.12
BSR1															1.00	0.93	-0.08	0.02
BSR2																1.00	-0.09	0.05
TreV																	1.00	0.17
SpuN																		1.00

Phenotypic correlation was measured as a Pearson's correlation coefficient (r) between the phenotypic values of the traits.

Supplementary Table S8. Estimates of the additive (σ_a^2) and dominance (σ_d^2) genetic and residual (σ_E^2) variances of the combined population.

	HarT	FruW	FruH	SugC	Aci	FruC	FruD	HeaR	WatC
σ_a^2	114.49	8195.01	0.37	0.38	0.04	1.55	0.00	0.01	0.01
σ_d^2	15.21	1586.17	0.11	0.08	0.01	0.33	0.00	0.00	0.00
σ_e^2	28.94	2182.34	0.16	0.23	0.01	0.34	0.00	0.01	0.01
h^2	0.72	0.68	0.58	0.55	0.68	0.70	0.33	0.34	0.29

Supplementary Table S9. Estimates of the additive (σ_a^2) and dominance (σ_d^2) genetic and residual (σ_E^2) variances of the parental population.

	HarT	FruW	FruH	SugC	Aci	FruC	FruD	HeaR	WatC	SWatC	FruS	Rust	Appear	Groove	BSR1	BSR2	TreV	SpuN
σ_a^2	179.50	8991.10	0.83	0.19	0.04	1.33	0.00	0.01	0.01	0.01	0.57	0.23	0.37	0.19	0.10	0.05	0.09	0.22
σ_d^2	56.91	3314.17	0.32	0.14	0.02	0.54	0.00	0.01	0.01	0.01	0.38	0.19	0.49	0.11	0.06	0.03	0.07	0.12
σ_e^2	78.79	4591.61	0.42	0.23	0.03	0.85	0.00	0.01	0.01	0.02	0.48	0.32	0.66	0.17	0.13	0.05	0.14	0.19
h^2	0.57	0.53	0.53	0.33	0.48	0.49	0.27	0.42	0.22	0.20	0.40	0.31	0.24	0.40	0.34	0.40	0.29	0.41

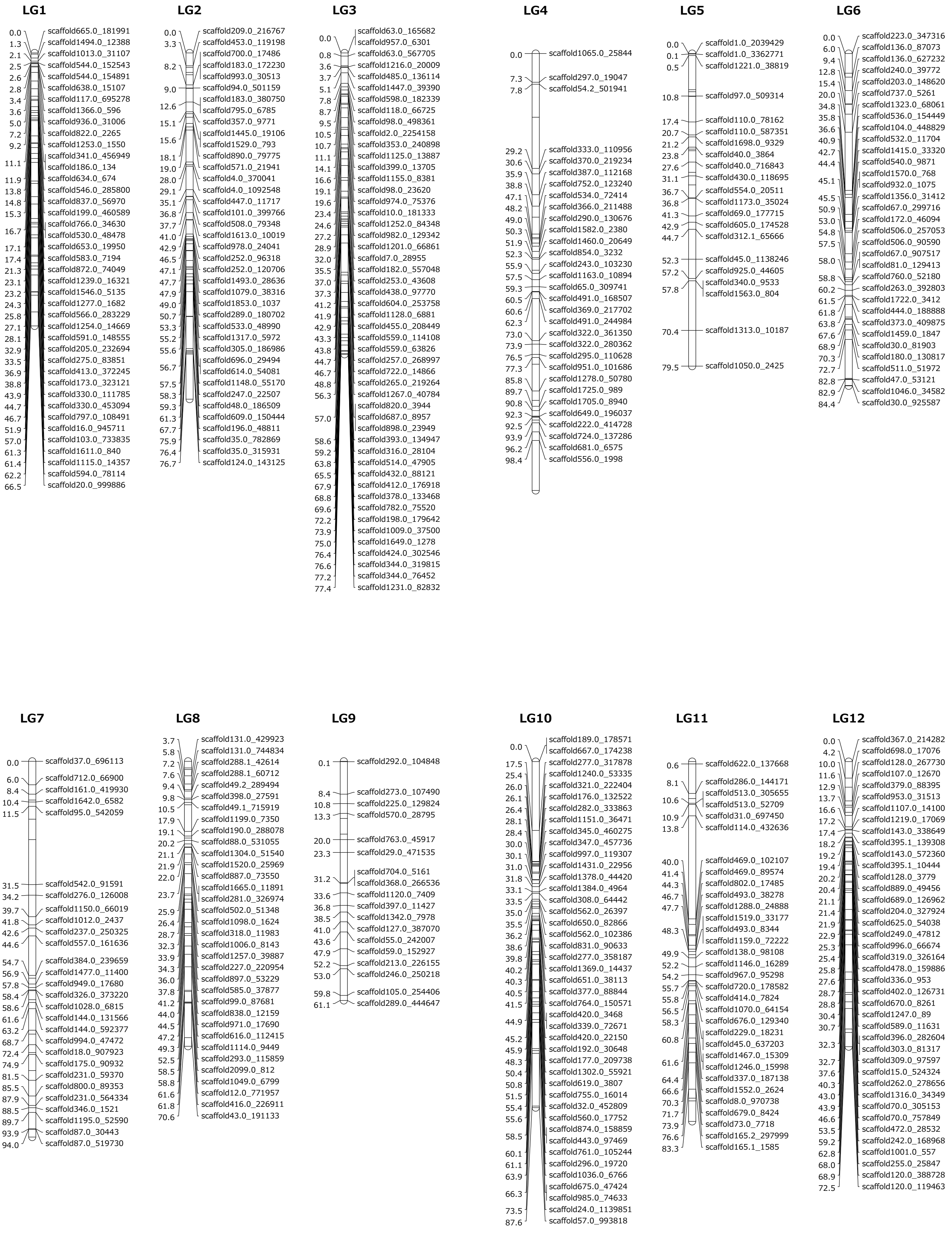

Supplementary Figure S1. Genetic linkage map for the F_1 family 523.
The map includes 563 SNPs on 17 LGs. Genetic distance (cM) is indicated on the left of each LG.

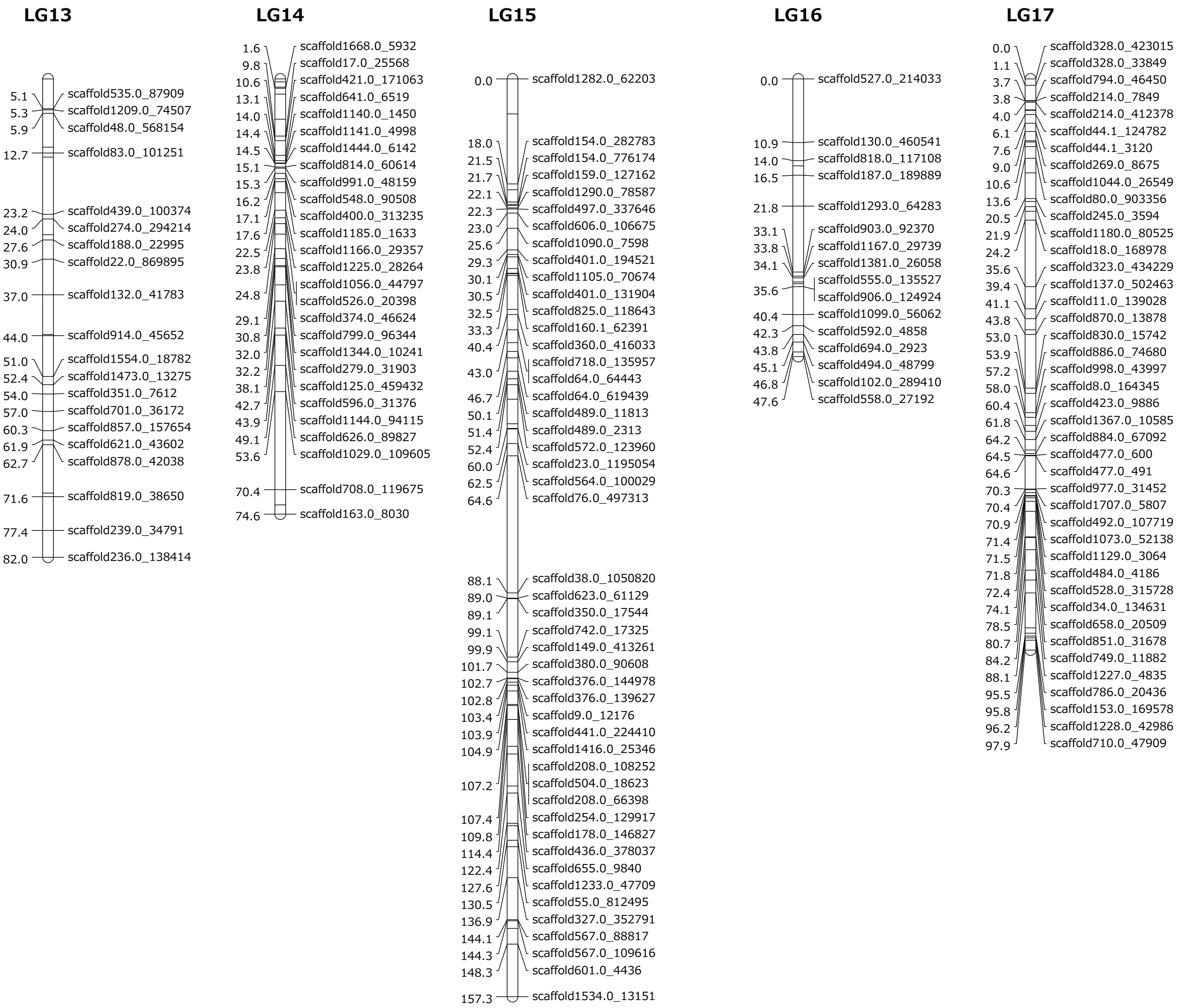

Supplementary Figure S1. Genetic linkage map for the F_1 family 523. (*Continued*)

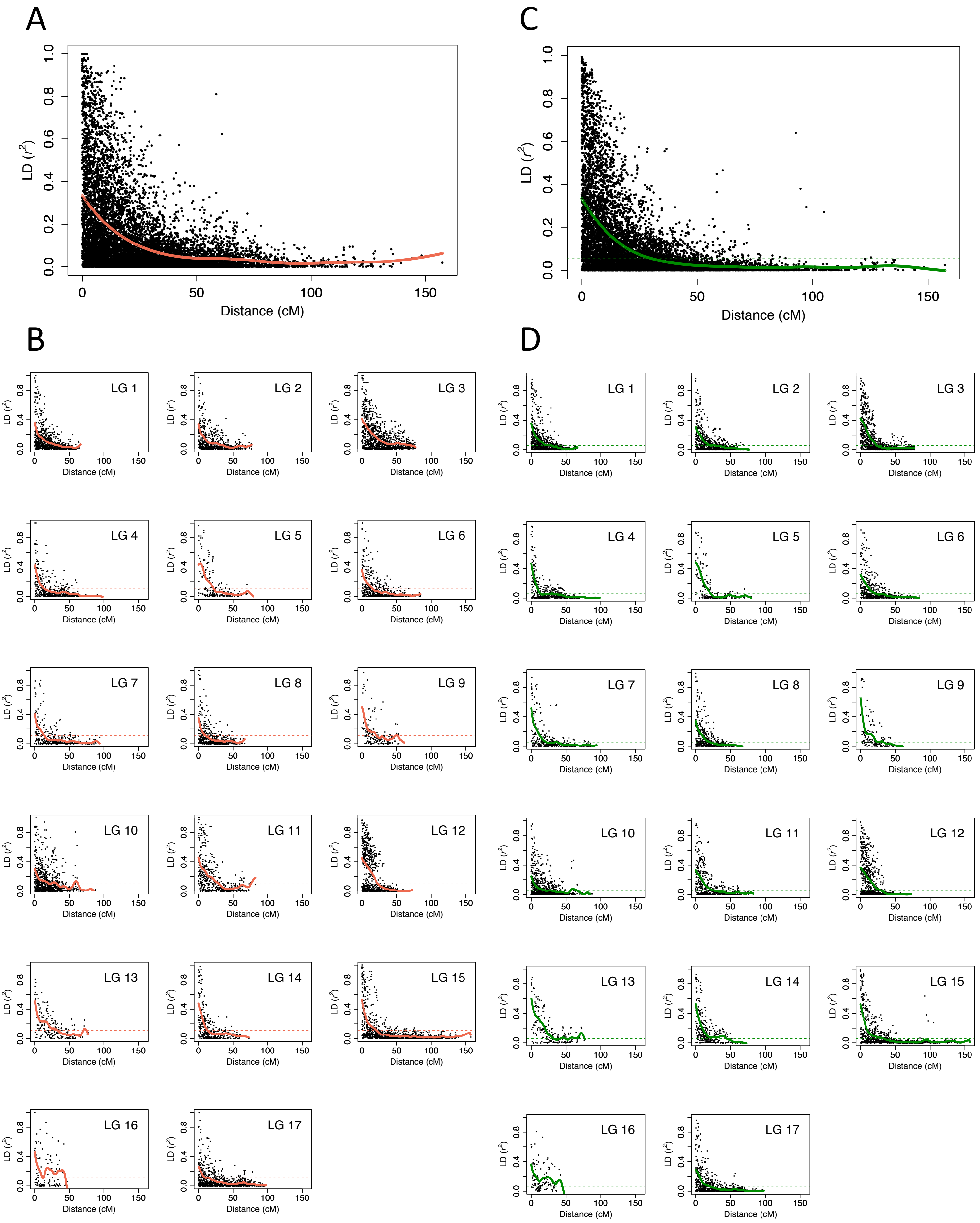

Supplementary Figure S2. LD values (r^2) between SNP pairs plotted against linkage map distances between the SNPs.

(A, B) Parental population; (C, D) combined parental and breeding populations;

(A, C) the entire genome; (B, D) individual chromosomes. Curves show local polynomial smoothed plots with kernel weight; horizontal dashed lines correspond to the baseline r^2 values based on the 95th percentile of the distribution of r^2 values between pairs of unlinked markers.

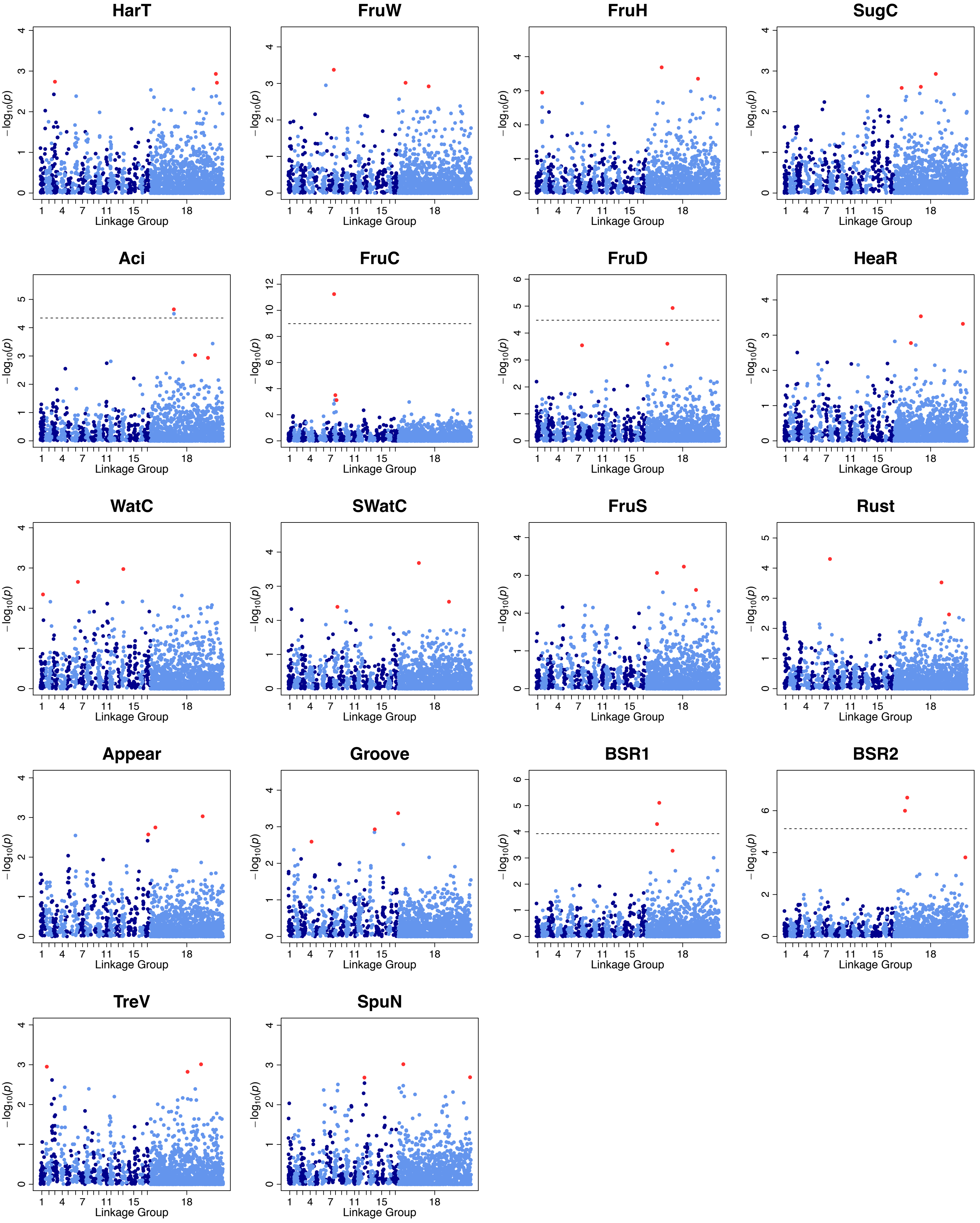

Supplementary Figure S3. Manhattan plots for 18 traits in single-locus GWAS using the parental population.

Dashed lines indicate a false discovery rate of 0.05. Linkage group 18 is a fictive linkage group for placing the SNPs not mapped on the 17 linkage groups. SNPs used for multiple linear regression (MLR) are shown in red (Figure 4C).

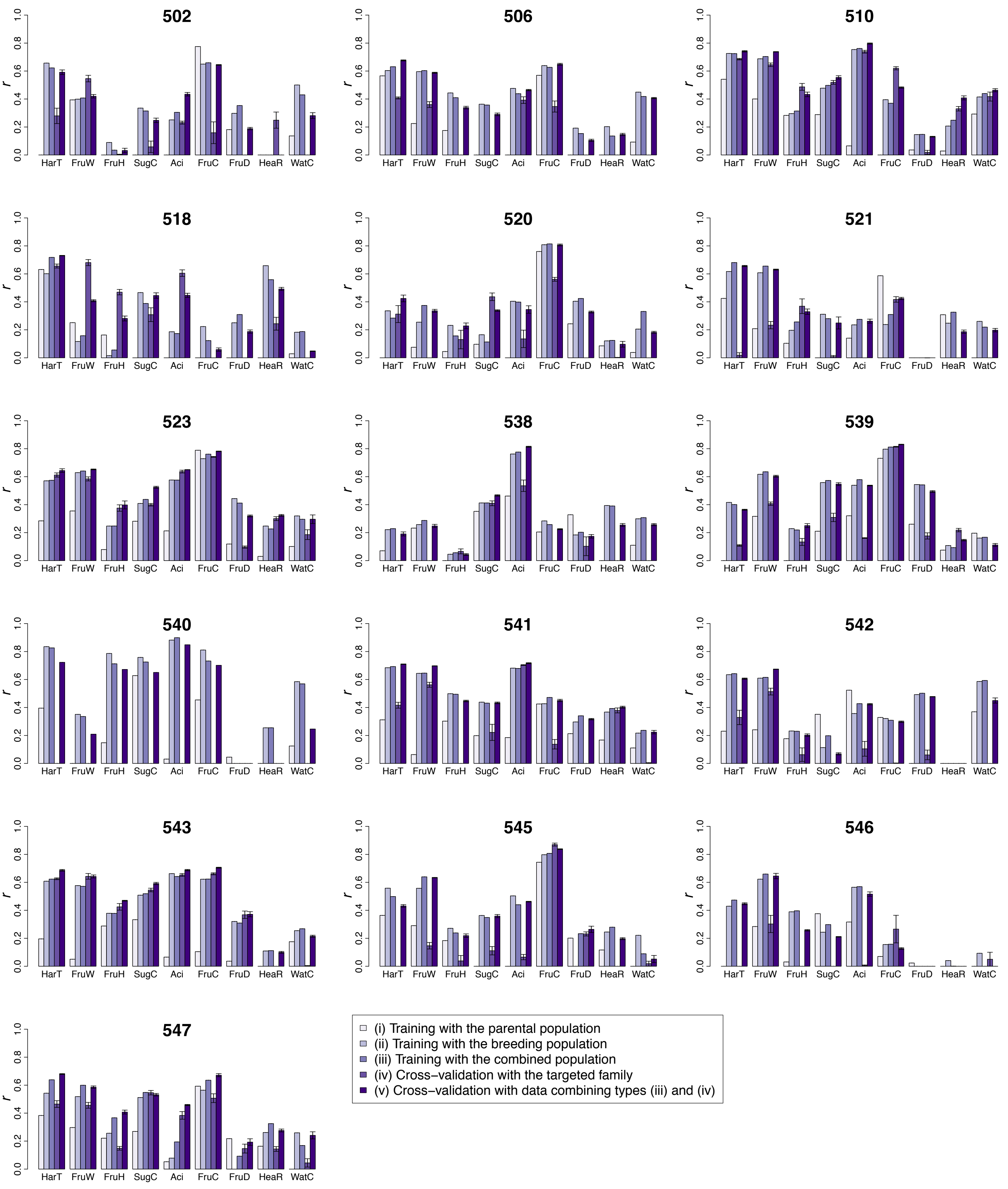

Supplementary Figure S4. Prediction accuracy (*r*) of five validation methods.
The prediction accuracy was evaluated for each family with the Pearson's correlation coefficient (*r*) between observed and predicted genotypic values.
Only the mean prediction accuracy of all the methods is shown.

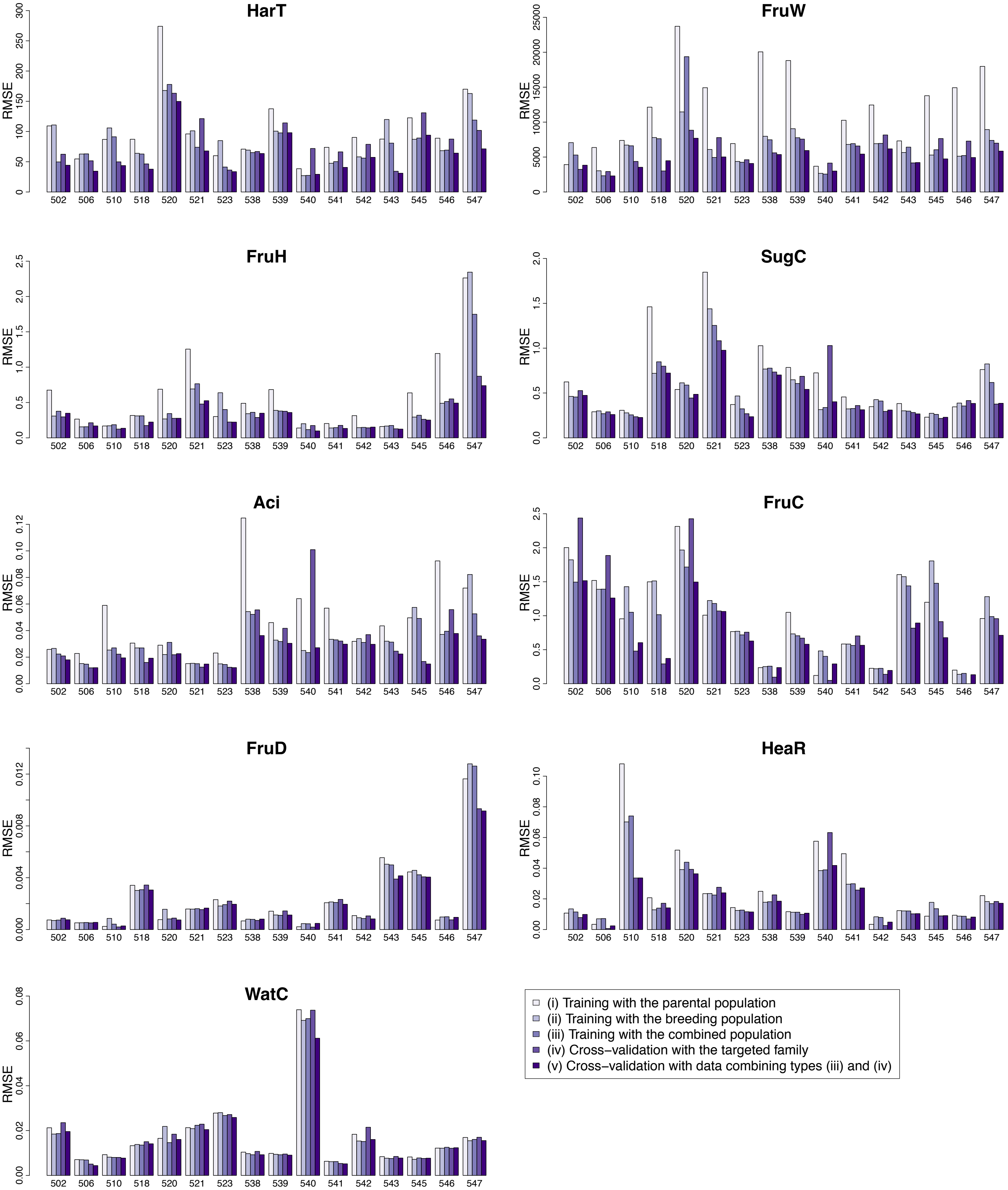

Supplementary Figure S5. Root-mean squared error (RMSE) of five validation methods.

The prediction accuracy was evaluated for each family with RMSE between observed and predicted values. Only the mean prediction accuracy of all the methods is shown.

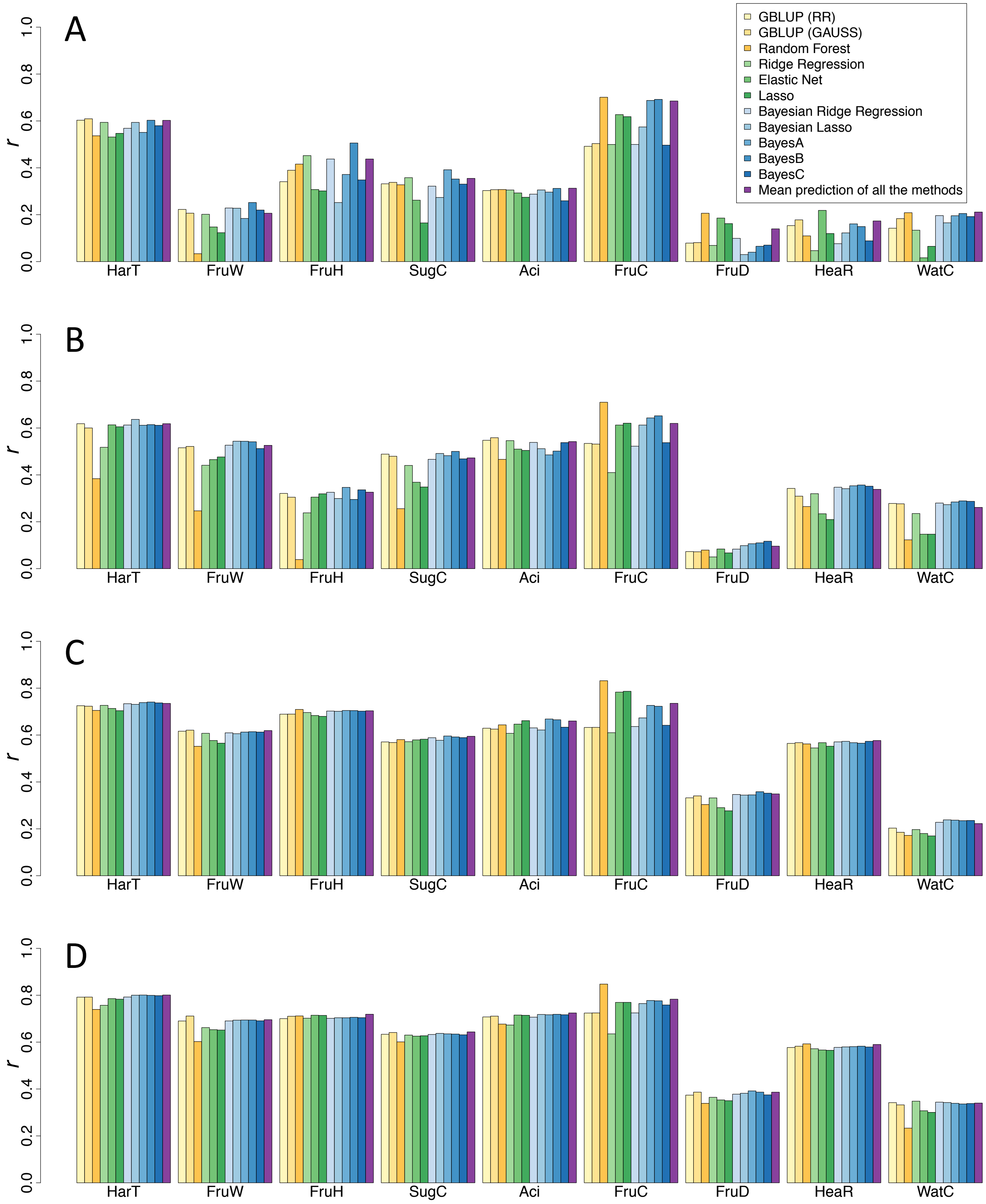

Supplementary Figure S6. Prediction accuracy of 12 methods for the breeding populations.

Prediction accuracy was measured as the Pearson's correlation coefficient (r) between predicted genotypic values and phenotypic values for all families combined.
RR: ridge kernel regression, GAUSS: Gaussian kernel regression.
Validation types: (A) (i); (B) (ii); (C) (iv); (D) (v) (see also Figure 4A).

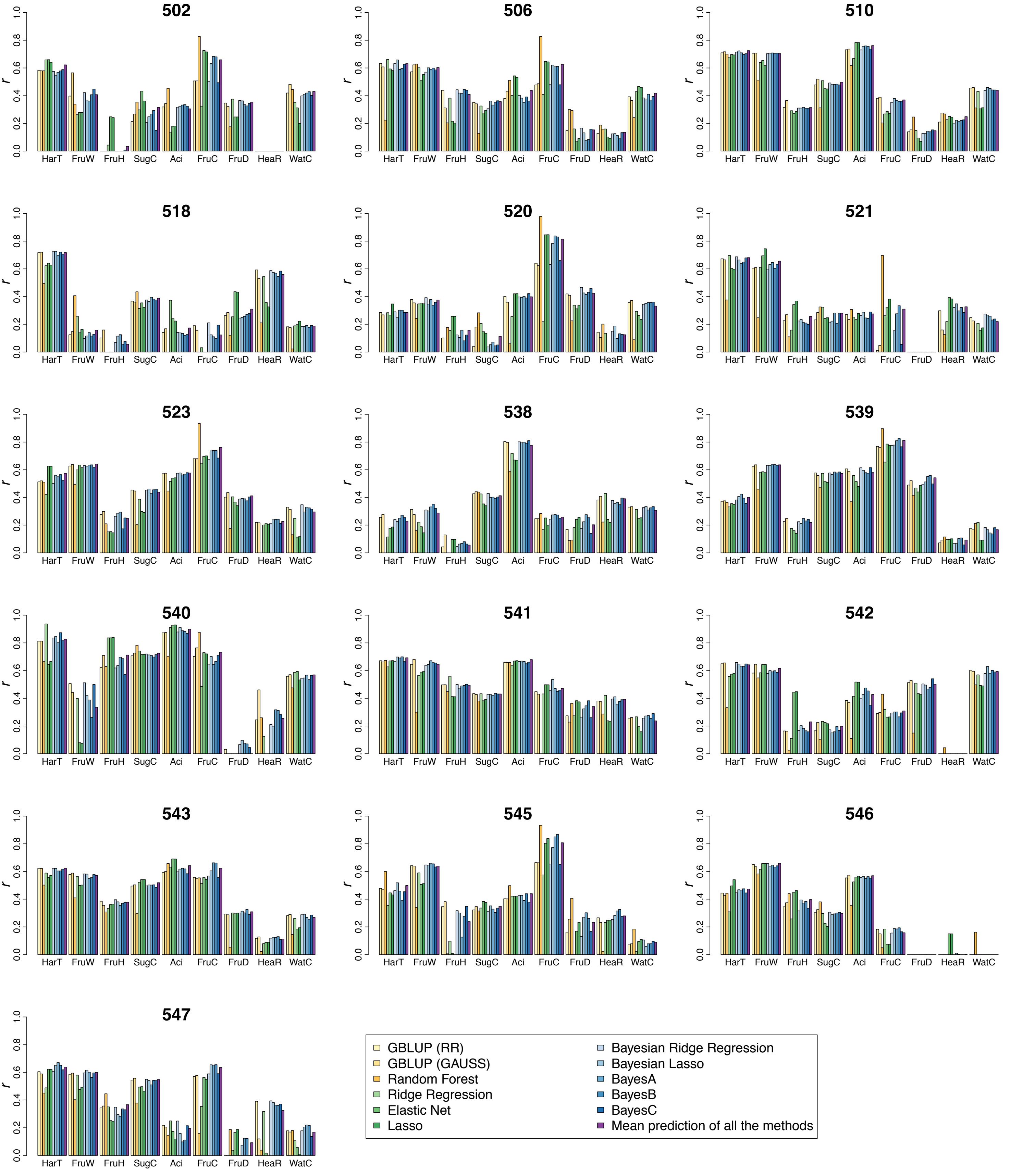

Supplementary Figure S7. Prediction accuracy (*r*) of 12 methods for each family of the breeding populations.

Only validation of type (iii) (Figure 4A) is shown.

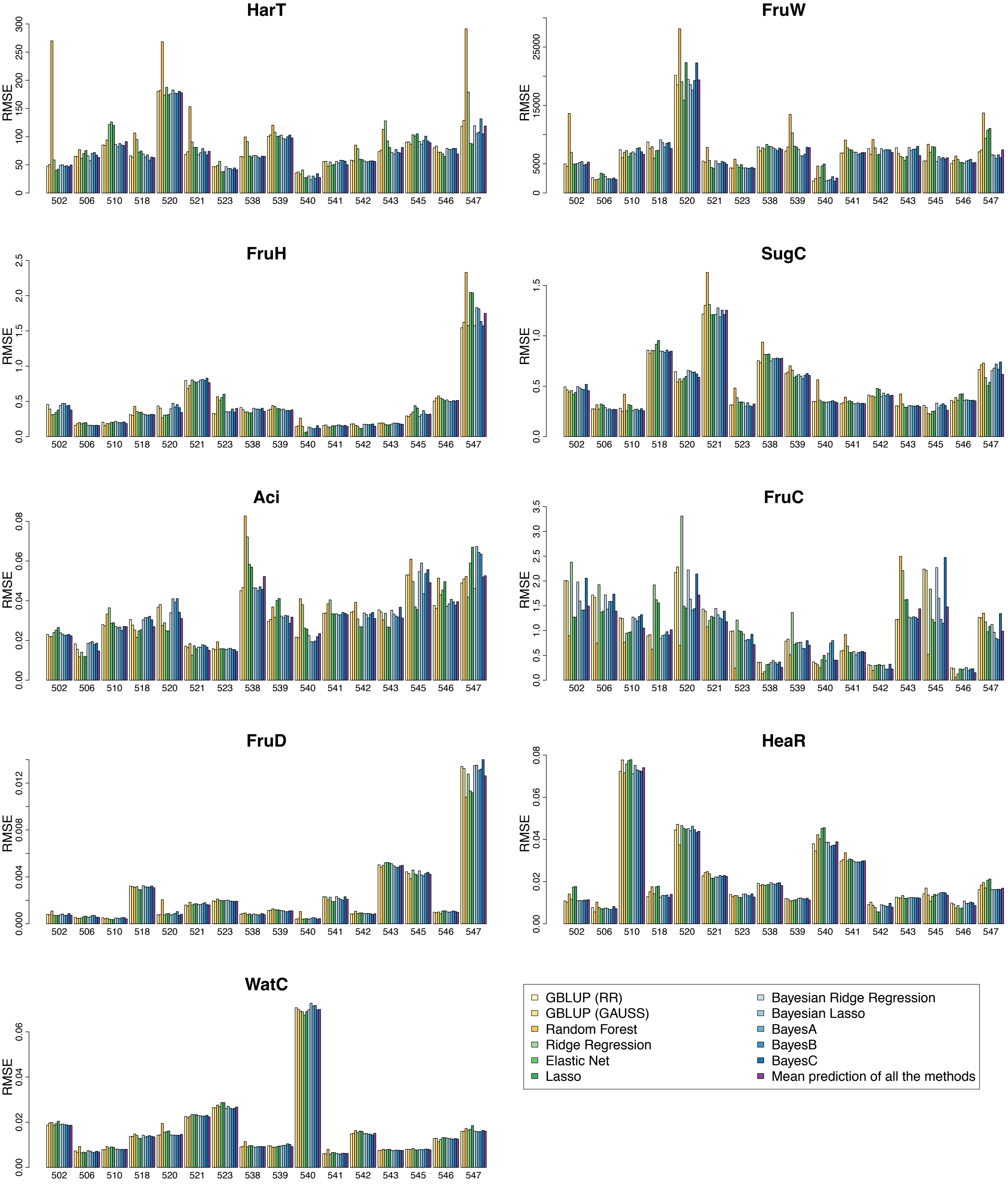

Supplementary Figure S8. Root-mean squared error (RMSE) of 12 methods for each family of the breeding populations.

The prediction accuracy was evaluated with RMSE between observed and predicted values. Only validation of type (iii) (Figure 4A) is shown.

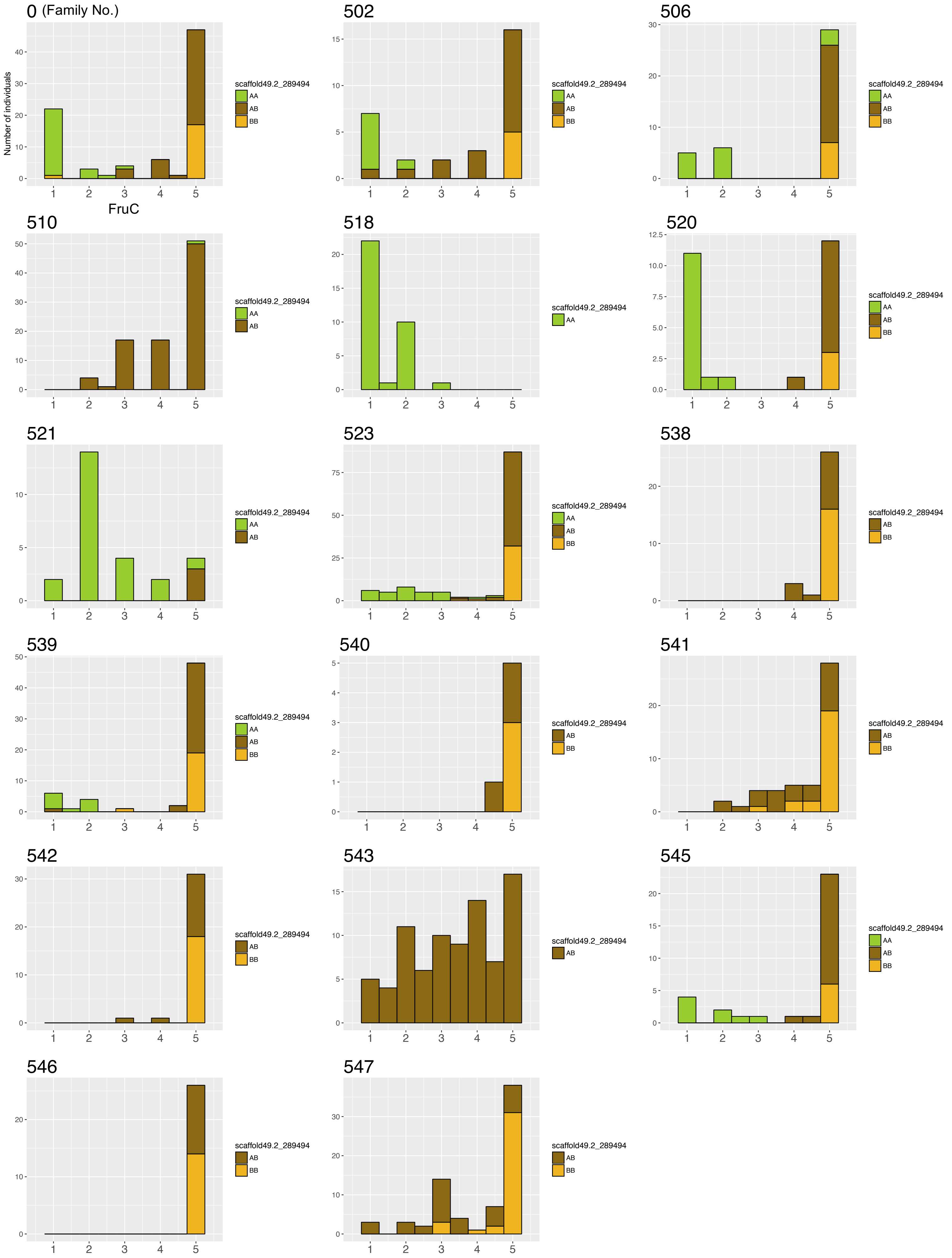

Supplementary Figure S9. Association between the most significant SNP allele detected in GWAS and phenotypic distribution of FruC in each family.

Parental population is shown as family 0; other numbers indicate breeding population families.

The *x*-axis shows the categorical score of FruC (Table 1).

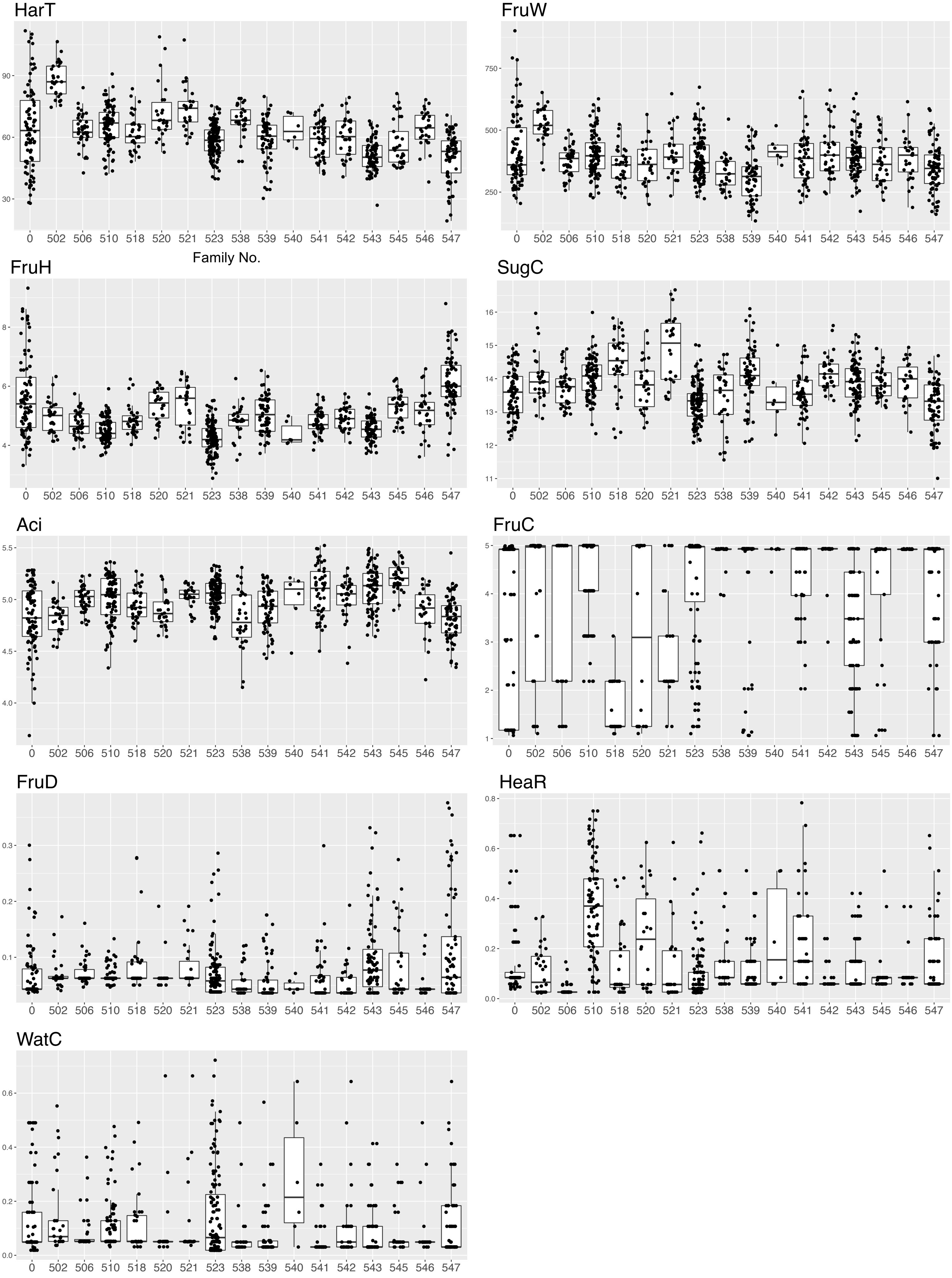

Supplementary Figure S10. Phenotypic variances of parental and breeding populations.
Phenotypic variations are visualized as jitter plots superimposed onto boxplots.
Parental population is shown as family 0; other numbers indicate breeding population families.

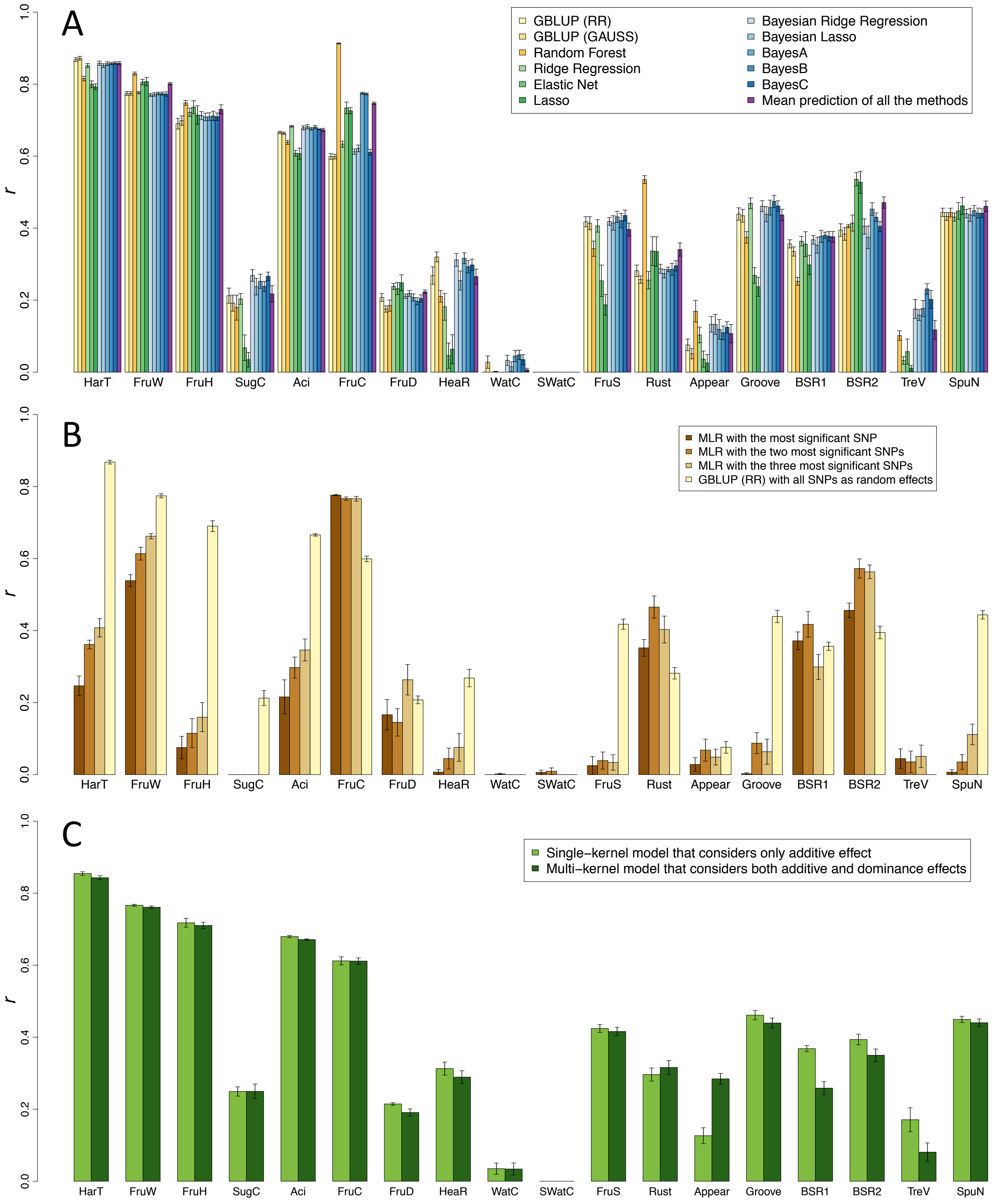

Supplementary Figure S11. Single-trait genomic prediction in cross-validation using the parental population.

Prediction accuracy was measured as the Pearson's correlation coefficient (*r*) between predicted genotypic values and phenotypic values. (A) Twelve methods were tested. RR: ridge kernel regression, GAUSS: Gaussian kernel regression. (B) Regression models based on the results of single-locus GWAS. One to three SNPs were selected that showed high $-\log_{10}(p)$ values in GWAS that used the parental population without genotypes targeted by genomic prediction. MLR: multiple linear regression. (C) Prediction models that considered only additive or both additive and dominance effects were used.

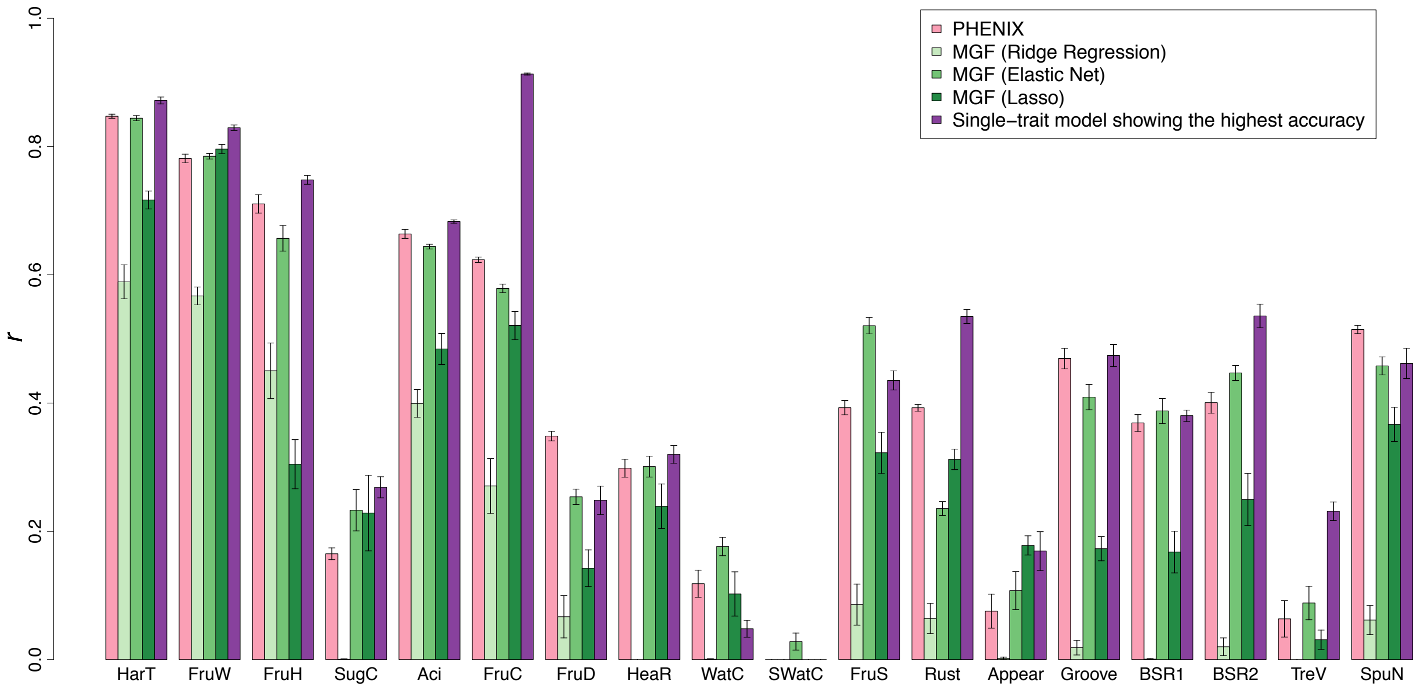

Supplementary Figure S12. Comparison of single- and multi-trait models in cross-validation using the parental population.

Prediction accuracy was measured as the Pearson's correlation coefficient (*r*) between predicted genotypic values and phenotypic values. PHENIX: Bayesian multivariate mixed model fitted via variational Bayes; MGF: multiresponse Gaussian family

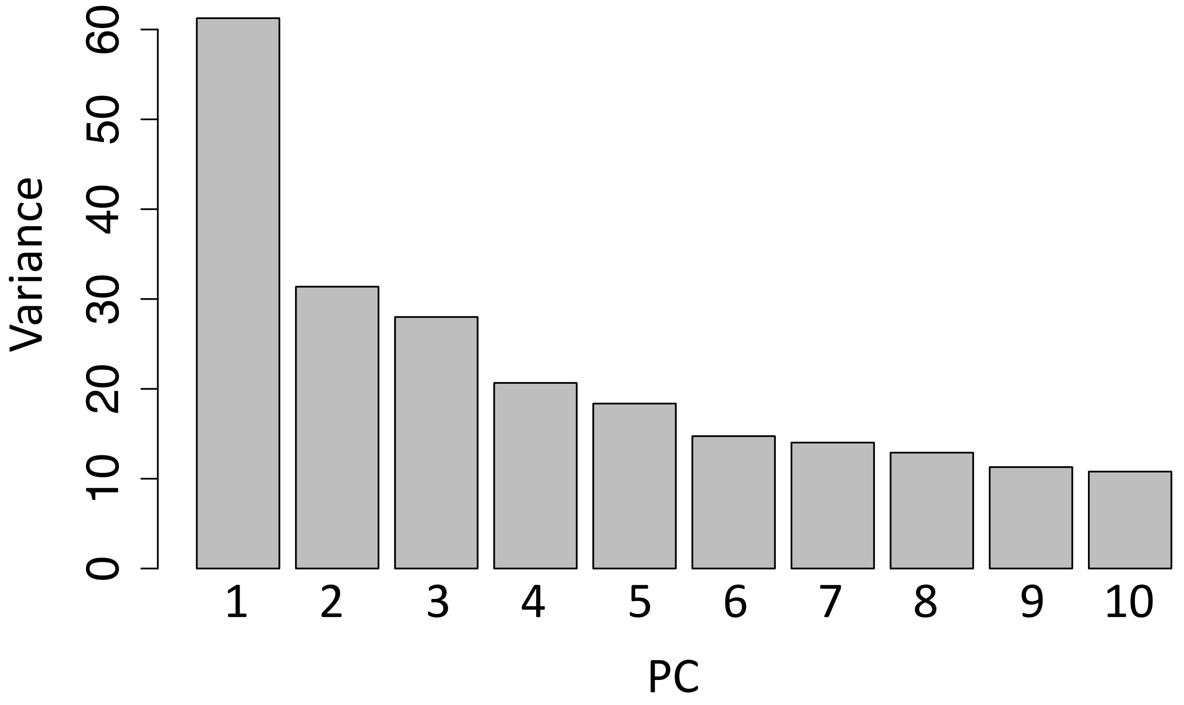

Supplementary Figure S13. Variances of principal component (PC) scores.
The variances were estimated from principal component analysis using the parental population.

Supplementary Methods

Next-generation sequencing and mapping

We used seven Japanese pear cultivars: 'Akizuki', 'Chojuro', 'Doitsu', 'Hosui', 'Niitaka', 'Nijisseiki', and 'Oushuu' for next-generation sequencing analysis. Genomic DNA of each cultivar was extracted from a young leaf using the DNeasy Plant Mini Kit (Qiagen) and used to construct a DNA library. Paired-end sequencing with an insert size of 500 bp was performed with a HiSeq 2000 system (Illumina Inc.) using 1 μg of genomic DNA for each sample.

Raw reads were trimmed to 90 bp and processed for quality control as follows. First, low-quality bases with quality scores below 20 were trimmed from both ends by using a home-made script. Second, the adapter sequences were removed with Cutadapt (Martin 2011) (option: -f fastq –e 0.1 –O 5 –m 20). The processed reads of each cultivar were mapped to the scaffold sequence of Chinese pear 'Dangshansuli' (Wu *et al.* 2013) with Burrows-Wheeler Aligner (BWA) 0.7.5a (Li and Durbin 2009) (option: aln –l 32 –R 30). The results were processed in SAMtools 0.1.19 (Li 2011) (option: view –S –b –f 2 –F 12 –q 20 –h) to exclude low-quality mapped reads. Local realignments were performed to correct misalignments with GenomeAnalysisTKLite 2.3-9 (McKenna *et al.* 2010) (option: -T IndelRealigner), and PCR duplicates were removed with Picard tools 1.92 (http://broadinstitute.github.io/picard) (option: MarkDuplicates.jar REMOVE_DUPLICATE=true).

The SNPs of each cultivar were called by SAMtools 0.1.19 (option: mpileup –C 50 –DSg -f) and GenomeAnalysisTKLite-2.3-9 (option: -T Unified Genotyper –I –genotype_likelihoods_model BOTH), and those with a read depth of 4–150× were used for further analyses.

SNP array design and genotyping

To design reliable SNP markers, we discarded SNPs meeting any of the following criteria: (1) other SNPs or indels were detected within the 80-bp flanking regions on either side of the SNP; (2) the 80-bp flanking sequences were aligned to two or more regions in the reference genome of Chinese pear 'Dangshansuli' by BLASTN search; (3) either of the 80-bp flanking regions included a repeat sequence; or (4) the read depth was extremely low or high (<20 or >60). Among the selected SNPs, those that were unsuitable for probe design because they had a SNP score under 0.7 (as calculated by the Illumina Assay Design Tool; Illumina Inc.) were discarded, and finally 1536 SNPs were selected for SNP genotyping array (Supplementary Data S1).

SNPs were genotyped using the Illumina GoldenGate Genotyping Assay (Illumina Inc.). The scanned data were analyzed with the Genotyping module (v. 1.9.4) of Illumina GenomeStudio v. 2011.1 software to generate genotype data for individuals. Clustering of SNPs was adjusted by eye when necessary. SNPs that had scores "GenTrain score" ≥ 0.4, "call freq" ≥ 0.85, "P-P-C errors" ≥ 2, and "minor freq" ≥ 0.01 were accepted.

Construction of genetic linkage maps

We used the F_1 family 523, which was the largest among the F_1 families of the breeding population (Supplementary Table S2). To construct an integrated map, we used the cross-pollination (CP) mode of JoinMap v. 4.1 software (Van Ooijen, 2006). Of the 1536 SNPs polymorphic in at least one parent ('Akizuki' or 373-55), 565 SNPs that showed clear segregation within the population were used (this includes marker configurations ab × cd, lm × ll, nn × np, ef × eg, and hk × hk). The 565 SNP markers were grouped with a minimum LOD score of 4.0 and a recombination frequency of 0.45. The regression mapping algorithm was used to build the linkage maps, and map distances were calculated according to the Kosambi mapping function (Kosambi, 1944). Finally, 563 of the 565 SNPs were mapped on the 17 LGs (Supplementary Figure S1), whereas 2 SNPs were not mapped on the 17 LGs. All 17 LGs were anchored to the reference genetic linkage maps of Japanese pear (Terakami *et al*. 2014).

References

- Kosambi, D. D. The estimation of map distances from recombination values. *Ann. Eugen.* **12,** 172–175 (1944).
- Li, H. A statistical framework for SNP calling, mutation discovery, association mapping and population genetical parameter estimation from sequencing data. *Bioinformatics* **27,** 2987–2993 (2011).
- Li, H. & Durbin, R. Fast and accurate short read alignment with Burrows-Wheeler transform. *Bioinformatics* **25,** 1754–1760 (2009).
- Martin, M. Cutadapt removes adapter sequences from high-throughput sequencing reads. *EMBnet.journal* **17,** 10–12 (2011).
- McKenna, A. *et al.* The genome analysis toolkit: A MapReduce framework for analyzing next-generation DNA sequencing data. *Genome Res.* **20,** 1297–1303 (2010).
- Terakami, S. *et al.* Transcriptome-based single nucleotide polymorphism markers for genome mapping in Japanese pear (*Pyrus pyrifolia* Nakai). *Tree Genet. Genomes* **10,** 853–863 (2014).
- Van Ooijen, J. W. JoinMap® 4, Software for the calculation of genetic linkage maps in experimental populations. *(Kyazma BV, Wageningen The Netherlands)* https://www.kyazma.nl (2006).
- Wu, J. *et al.* The genome of the pear (*Pyrus bretschneideri* Rehd.). *Genome Res.* **23,** 396–408 (2013).
